# Restricted Dispersal in the Late Successional Forest Tree Species *Nothofagus Pumilio*: Consequences Under Global Change

**DOI:** 10.1002/ece3.71002

**Published:** 2025-05-23

**Authors:** C. Soliani, J. Sekely, C. Zamora‐Ballesteros, K. Heer, O. Lepais, V. Mondino, L. Opgenoorth, M. Pastorino, P. Marchelli

**Affiliations:** ^1^ INTA Bariloche, Instituto de Investigaciones Forestales y Agropecuarias Bariloche IFAB (INTA‐CONICET) Bariloche Argentina; ^2^ Eva Mayr Stihl Professorship for Forest Genetics Albert‐Ludwigs Universität Freiburg Freiburg Germany; ^3^ Plant Ecology and Geobotany Philipps‐Universität Marburg Marburg Germany; ^4^ Univ. Bordeaux, INRAE, BIOGECO Cestas France; ^5^ INTA Esquel Esquel Argentina

**Keywords:** direct method, gene flow, pollen dispersal, seed dispersal, seed donors, spatial genetic structure

## Abstract

Plants rely on gamete dispersal to ensure the inheritance of their genes. Gene flow, mediated by pollen and seed dispersal, also fosters species' cohesion across space, facilitates population migration, and influences local adaptation. *Nothofagus pumilio* is an ecologically important wind‐dispersed tree species of the Patagonian Andes. We aim to uncover its current and historic effective dispersal distances and characterize its fine‐scale genetic structure. In a naturally monospecific stand of *N. pumilio* , we sampled 200 adults and 400 seedlings. Using a modern sequencing approach (SSRseq), we developed 15 nuclear microsatellite markers for genotyping and used them to characterize genetic diversity and fine‐scale genetic structure. We estimated dispersal distances using direct methods (i.e., neighborhood models) and indirect (i.e., inferred from fine‐scale spatial genetic structure). Short average seed and pollen dispersal distances were estimated (δ_s_ = 13.33 m and δ_p_ = 24.08 m respectively), but the fat‐tailed distribution of dispersal kernels also suggests some immigration and long‐distance dispersal events. Indirect estimates (*σ* ^*2*^_*g*_ = 21.62) are closely aligned with direct estimates. The majority of seedlings (84%) could be assigned to at least one sampled adult within the plot, and these seedlings were produced by just 43% of sampled adults. Reproductive success was significantly associated with seed donors' diameters at breast height. *N. pumilio* 's distribution expansion capacity may be limited by short seed dispersal distances, especially in the context of global change. Natural and assisted migration actions should be prioritized to mitigate future change effects.

## Graphical abstract

We aim to uncover the current and historic effective dispersal distances and characterize fine‐scale genetic structure in the forest tree species Nothofagus pumilio. Short seed dispersal distances mean *N. pumilio* distribution expansion capacity may be limited, especially in the context of global change. Natural and assisted migration actions should be prioritized to mitigate future change effects.

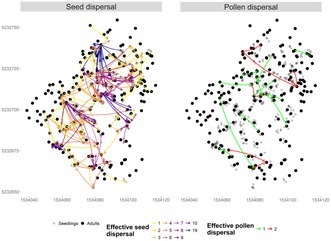

## RESUMEN

Para garantizar la herencia de sus genes, las plantas dependen de la dispersión efectiva de sus gametas. El flujo genético, mediado por la dispersión del polen y las semillas, promueve la cohesión en el espacio a nivel intra‐específico, facilita la migración de las poblaciones e incide en la adaptación local. *Nothofagus pumilio* es una especie arbórea de gran importancia ecológica de los bosques Andino –Patagónicos que se dispersa por el viento. En este trabajo, nuestro objetivo es conocer sus distancias de dispersión efectivas, actuales e históricas, y caracterizar su estructura genética a pequeña escala. En un rodal monoespecífico de *N. pumilio*, tomamos muestras de 200 adultos y 400 plántulas. Utilizando una moderna metodología de secuenciación (SSRseq), desarrollamos 15 marcadores microsatélites nucleares para el genotipado de los individuos y los utilizamos para caracterizar la diversidad y la estructura genética a pequeña escala. Estimamos las distancias de dispersión utilizando métodos directos (por medio de modelos de vecindad) e indirectos (inferidos a partir de la estructura genética espacial a pequeña escala). Estimamos las distancias medias de dispersión de las semillas y el polen (δs = 13.33 m y δp = 24.08 m respectivamente), que indicaron una dispersión efectiva a corta distancia. La distribución de los núcleos de dispersión sugiere además la ocurrencia de algunos eventos de inmigración y dispersión a larga distancia, o bien desde las inmediaciones de la parcela. Las estimaciones indirectas (σ2g = 21.62) coinciden con las directas. Logramos asignar al menos un progenitor a la mayoría de las plántulas (84%), que fueron producidas por sólo el 43% de los adultos muestreados. También se midió la altura y el diámetro a la altura del pecho (DAP) de los adultos, y el éxito reproductivo se asoció significativamente con el DAP de los árboles que actuaron como donantes de semillas (flores femeninas). Las cortas distancias de dispersión de las semillas sugieren que la capacidad de expansión de la distribución de *N. pumilio* puede ser limitada, especialmente en el contexto del cambio global. Proponemos que deberían priorizarse las acciones de migración natural y/o asistida para mitigar los futuros efectos del cambio climático.

## 1 Introduction

Dispersal is a critical process in plant evolution (Cruzan and Hendrickson 2020). Effective dispersal distance of pollen and seeds is a decisive factor (Nathan et al. 2012) because it determines how far alleles and individuals can move through generations, which is known as gene flow. Dispersal distance is also tightly linked to several life‐history traits (e.g., fecundity, pollen and seed dispersal mode, seed size, seed shape) and is conditioned by various abiotic factors, including environmental variation (Proença‐Ferreira et al. 2023) and topographic barriers (e.g., Hernández‐Leal et al. 2022; Millerón et al. 2008). Gene flow is critical for maintaining genetic continuity among populations, ensuring species' persistence despite local extinction risk (Ronce 2007), and shaping spatial patterns of genetic diversity. Disturbances such as glaciations can restrict gene flow and lead to geographic isolation of populations (e.g., Sérsic et al. 2011; Magri et al. 2006), which can profoundly impact plant population dynamics (e.g., Petit et al. 2004) and create spatial population structure. However, subsequent gene flow following such disturbances can facilitate species persistence and continued evolution. Finally, the balance between gene flow and selection can affect patterns of local adaptation (Sork 2016). When dispersal distances are short and selection is strong, local adaptation patterns may be enhanced, but if gene flow is strong enough to swamp alleles under selection, local adaptation may be reduced (Slatkin 1978). Thus, understanding effective dispersal distances as a component of gene flow can elucidate many parts of species' evolution.

Gene flow is mediated by pollen and seed dispersal in trees (Savolainen et al. 2007; Sork 2016) and tends to be spatially limited, although rare long‐distance dispersal events can play a major role in large‐scale population dynamics (Nathan et al. 2008; Wu et al. 2023). In wind‐dispersed tree species, smaller pollen grains generally have dispersal distances that are orders of magnitude greater than the heavier seeds (Ennos 1994). This is particularly relevant because pollen and seed dispersal have different evolutionary impacts: while pollen can only facilitate allele movement, seeds can also mediate population migration and colonization. Thus, gene flow has the potential to introduce new alleles to declining populations or individuals to new habitats. These characteristics are especially critical in the face of climate change, which is expected to have diverse impacts on tree populations, including changes in phenology (Vitasse et al. 2021), microhabitat modifications (De Frenne et al. 2021) that can affect local selection pressures, and changes in effective population sizes (Santos‐del‐Blanco et al. 2022). In the case of anemophilous (i.e., wind‐dispersed) species, global wind circulation has a large‐scale influence on spatial genetic patterns (i.e., genetic diversity, genetic differentiation, and asymmetric gene flow) (Kling and Ackerly 2021), and ongoing climate change has the potential to alter global wind patterns and thereby patterns of gene flow. Understanding past and contemporary patterns of gene flow could help forecast the population's potential to cope with future change.

*Nothofagus pumilio* (Poepp. & Endl.) Krasser is a monoecious, strictly outcrossing, and wind‐dispersed native tree species of the Temperate Forests of South America (Veblen et al. 1996). It predominantly inhabits the Cordillera de los Andes in Chile and Argentina, from 36° S to 55° S, and its 2200 km‐long distribution range is the largest among the South American *Nothofagus* species. *Nothofagus pumilio* is known for its cold tolerance, and it dominates forests at higher elevations, where it forms naturally monospecific stands up to the alpine treeline. In high‐elevation areas where low temperatures and frosts prevail, it typically grows in shrub formation, while at lower elevations, individual trees grow in arboreal form and can exceed 35 m in height (Tortorelli 1956). *Nothofagus pumilio* experiences irregular reproductive cycles and high mortality rates at early life stages, both common features in tree species (Petit and Hampe 2006), which may impact recruitment and seedling establishment (e.g., Gerzabek et al. 2020). Reproduction is predominantly conducted through seed generation (Soliani et al. 2021), although prior studies at the edge of second‐growth forests have described a variable proportion of selfing, as determined by the presence of half‐ to full‐sibling individuals (Till‐Bottraud et al. 2012; Fajardo et al. 2016). Regeneration also varies across time and space. *Nothofagus pumilio* exhibits masting behavior, with synchronous interannual cycles of abundant seed production that are associated with higher germination rates and viability (Donoso Zegers 1993; Koenig 2021). Spatially, regeneration may involve stand replacement after avalanches and increasingly abundant wildfires (Kitzberger et al. 2022) or smaller events following localized disturbances like the natural falling of over‐mature trees (Veblen et al. 1996; Donoso Zegers 2006). *Nothofagus pumilio* seeds have time‐limited viability, typically around 1 year (León‐Lobos and Ellis 2005; Urretavizcaya et al. 2022), and therefore do not form permanent seed banks (Cuevas and Arroyo 1999). However, seedling banks can persist for many years below the adult generation while awaiting suitable conditions for further growth and development (Cuevas and Arroyo 1999; Martínez Pastur et al. 2011). Therefore, it is critical to understand seed and pollen flow dynamics in order to understand the species' natural regeneration capabilities and limits.

Although various aspects of the reproductive biology of *N. pumilio* have been extensively studied in situ, such as flower and seed production (Cuevas 2000; Martínez Pastur et al. 2008), flower phenology (Rusch 1993), and seed viability and germination capacity (González et al. 2006), many questions remain. The effective dispersal distances of pollen and seeds have not yet been evaluated in this species, although limited dispersal has been observed in other *Nothofagus* species at stand‐level spatial scales (e.g., Sola et al. 2022). Seed dispersal distances of *N. pumilio* have been estimated using seed traps, and results suggested they are predominantly dispersed only short distances from the donor tree (Rusch 1993). Meanwhile, pollen dispersal has not been formally analyzed. A previous genetic structure analysis of *N. pumilio* adults along an elevational gradient showed significant fine‐scale spatial genetic structure, especially at short distances (Mathiasen and Premoli 2013). However, there is still a need for multigenerational fine‐scale spatial genetic structure analysis to understand seed and pollen dispersal over longer timeframes. Furthermore, since gene flow is a critical factor in the equation of overall local adaptation, characterizing patterns can help elucidate possible species' evolutionary response to future change. This study aims to assess current and historic gene flow in the species, using parentage analysis and fine‐scale spatial genetic structure analysis respectively, as an evolutionary force shaping the population genetic structure throughout its life history. Specifically, we address two questions. First, what are the effective pollen and seed dispersal distances in *N. pumilio* ? Based on data from other wind‐dispersed species (e.g., Semizer‐Cuming et al. 2021), we expect effective pollen dispersal distances to be greater than seed dispersal distances. Furthermore, limited seed dispersal has been seen in related species (e.g., Sola et al. 2020) and is likely to result in fine‐scale spatial structuring. Second, do fitness‐related phenotypes in adults influence reproductive success? We expect larger trees tend to produce greater amounts of pollen and seeds and thus contribute a larger percentage of subsequent generations, as has been observed in other *Nothofagus* species (Sola et al. 2020, 2022). Addressing these questions could provide important information about the species' potential to track its ecological and climatic niches under climate change.

## 2 Material and Methods

### 2.1 Study Site and Sampling

**FIGURE 1:**
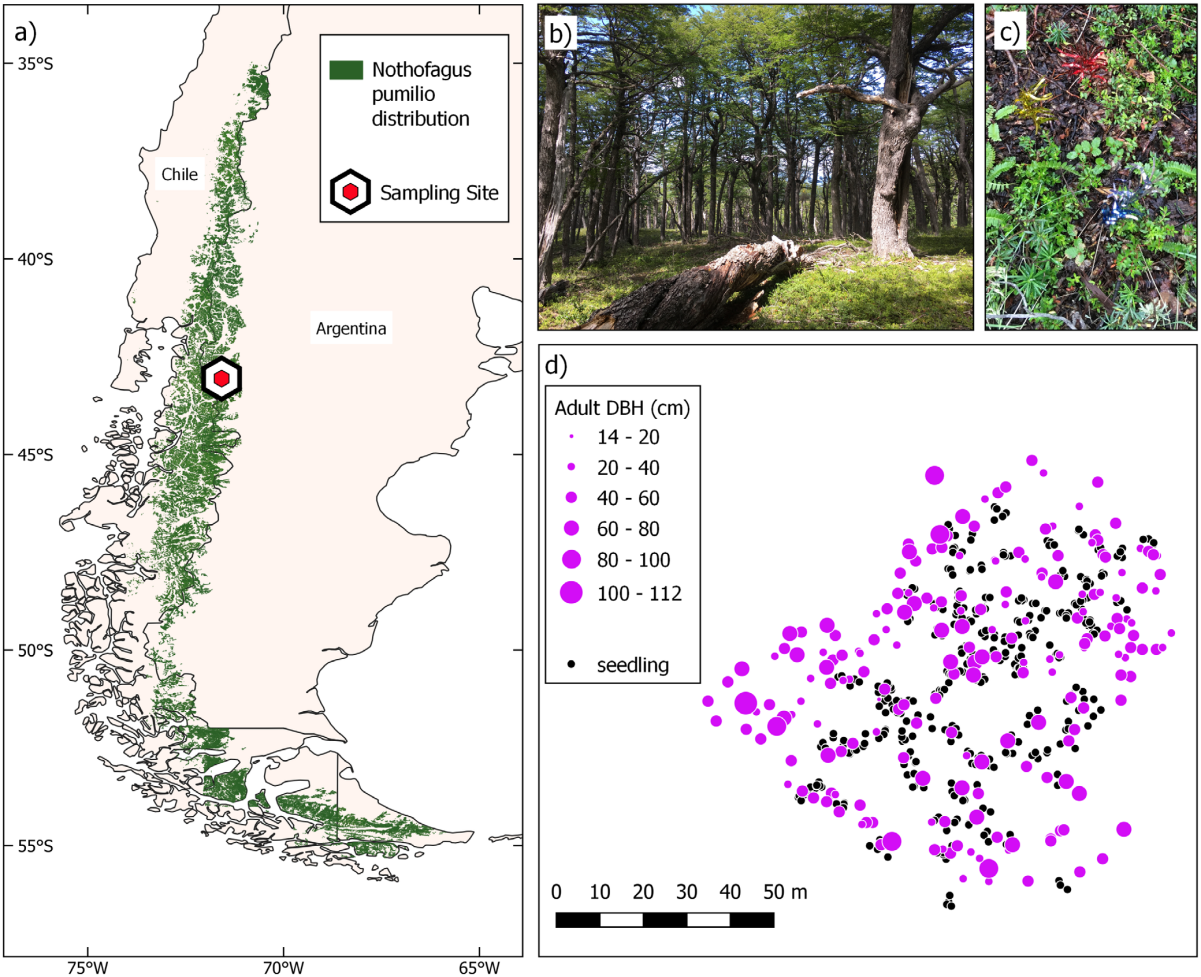
Characteristics of the *Nothofagus pumilio* study plot and the sampled adults and seedlings. (a) species distribution range and sampling site location; illustrative photos of (b) adult trees and overall stand characteristics and (c) site ground cover and 3 sampled seedlings, which are marked with colored stakes for georeferencing; (d) spatial configuration of sampled seedlings (black dots) and adults (purple dots), with adult dot diameters indicating diameter at breast height (DBH) in centimeters. Figure created with QGIS 3.22.4.

The study site is located in Parque Nacional Los Alerces near Trevelin, Chubut Province, Argentina (−43.05811°, −71.58161°). The site is a typical forest stand, located within a continuous and uneven‐aged forest that comprises a naturally single‐species stand of *Nothofagus pumilio*. It is on the leeward side of the Andes. The site is relatively flat and has an average elevation of 1230 m above sea level (range 1225–1240 m asl), which is near the middle of the species' elevation distribution at this latitude. Although the site is near the eastern edge of the species' distribution, climate data from two sources indicate that the chosen site exhibits moderate temperature and precipitation conditions in relation to the overall climate space occupied by the species (Sekely et al. 2024). First, historical empirical records from the nearest meteorological station (INTA Trevelin Station, ~380 m a.s.l.) show a mean annual temperature of 9.9°C and annual precipitation of 975.8 mm, although the study site is at a much higher elevation and therefore is likely colder and wetter. Second, historic data for the sampling site, extracted from the online climate repository CHELSA (v 2.1, Karger et al. 2017, 2018), show a mean annual temperature of 5.9°C and annual precipitation of 1866 mm, although CHELSA models have been shown to overestimate precipitation in the Patagonian Andes (Fierke et al. 2024). At the time of sampling, most regeneration within the plot consisted of yearlings (i.e., one‐year‐old seedlings, with an average height of 6 cm) and a few scattered older seedlings. Undergrowth is generally limited (Figure 1b,c) and there is no *Chusquea culeou* E. Dev., a bamboo that strongly competes with *N. pumilio* regeneration in adjacent forests at lower and warmer elevations (Veblen 1982).

Samples were collected in the austral summer of 2020 (February). Beginning from an arbitrary central point and working progressively outwards in a circular manner, we exhaustively sampled every healthy adult tree of reproductive age until we reached our target of 200 individuals. Next, we haphazardly selected between 1 and 5 yearlings that were positioned directly underneath one of the sampled adults' crowns. In total, we collected 400 seedlings, located between 0.2 and 5.1 m from the closest adult trunk (average distance 1.7 m). Georeferencing was performed with a Haglöf Vertex Laser Geo device (Långsele, Sweden). Adult trees were recorded with the GPS function, then relative positions of the 1–5 seedlings were recorded with the Map Target function by placing the ultrasonic transponder at the base of each seedling and measuring from the adult's trunk. The total dimensions of the plot were approximately 100 m by 80 m (0.7 ha; Figure 1). Heights of seedlings and adults were recorded using a ruler and the Haglöf Vertex Laser Geo, respectively. Diameter at breast height was measured for adults. Sampled adults had an average height of 16 m (range 6–27 m) and diameter at breast height of 47 cm (range 14–112 cm) (Figures 1, S1). Leaf material was collected for all seedlings and most adult trees. In some adults, leaves were unobtainable due to tree height (*n* = 35 adults); in which case, a cambium sample was collected instead using a Haglöf increment borer. All fresh samples were immediately placed in empty tea bags and stored with silica gel to remove moisture.

### 2.2 DNA Extraction

Depending on the sample, we used either 20–30 mg of dried leaf material or 40 mg of cambium that had been cut into thin slices with a razor blade and then frozen at −80°C overnight to make the material brittle. Due to the high polyphenol content of *N. pumilio* leaves, we followed the DNA extraction protocol of Inglis et al. (2018). First, phenols were removed from leaf samples using a sorbitol‐based pre‐wash, followed by a CTAB‐based extraction method. For cambium samples, we omitted the sorbitol pre‐wash and simply began with CTAB extraction. DNA concentration was measured using QUBIT 1.0. In addition, a random subset of extracted DNA samples was quality checked by PCR using *N. pumilio* specific primers (Soliani et al. 2010) to ensure that there was sufficient DNA for replication during genotyping.

### 2.3 Genotyping

We employed a new next‐generation sequencing approach to genotype microsatellite markers using high‐throughput sequencing (SSRseq). While classical microsatellite analysis only determines the fragment length, this newer approach provides full sequence information, including allelic variation that would not be detected if it does not affect the length (Lepais et al. 2020). Both nuclear microsatellite (“SSR”) and chloroplast (“cpSSR”) markers were targeted, ideally to distinguish between paternal and maternal gene flow, since chloroplast markers are maternally inherited. For SSR marker development, we followed the protocol of Lepais et al. (2020). First, shotgun sequencing based on pooled DNA from three individuals was conducted to identify suitable SSR loci. Illumina Miseq sequencing resulted in 1.8 million 2 × 300 bp reads (ENA accession number: ERR13472835). Out of these, 1.2 million reads were merged using BBmerge (Bushnell et al. 2017). Using QDD software (Meglécz et al. 2014), a total of 26,709 nuclear SSRs were identified, of which 7741 had designed primers. Primers for 60 loci were developed (Data S1), and 45 that gave a successful amplification were kept in the final multiplexed PCR. Raw sequences were converted to genotypes accounting for all polymorphisms (microhaplotypes) using a pipeline (Lepais et al. 2020) integrating FDSTools (Hoogenboom et al. 2017). After initial tests, 30 of the nuclear loci were selected for final genotyping, and 17 produced reliable results (genotyping error < 3% and < 30% missing data). For chloroplast analysis, we used the software NOVOplasty version 3.7.2 (Dierckxsens et al. 2017) to assemble a portion of the *Nothofagus* chloroplast genome using the rbcL gene (GenBank accession number: L13360.2) as a seed, resulting in one contig of 71,788 bp (Data S1). Mapping the raw reads to this contig, 17 mononucleotidic cpSSR, substitutions, or insertion‐deletions (indels) were identified. For 19 markers, primers were designed with BatchPrimer3 (You et al. 2008) and used to amplify and genotype these markers (Data S1) by sequencing them together with the nuclear markers. Amplicons were indexed using Nextera combinational barcodes and sequenced in Illumina Miseq V2 2 × 150 bp reads configuration. The polymorphism of the chloroplastic markers was very low and only 3 reliable polymorphic markers were identified.

### 2.4 Data Analysis

Standard genetic parameters (number of alleles (N_A_), effective number of alleles (A_E_), private alleles (Ap), observed and expected heterozygosity (H_O_ and H_E_)) were estimated for each generation (adults and seedlings) with GenAlEx v. 6.5 (Peakall and Smouse 2012). Allelic richness (A_R_), after rarefaction to an equal sample size (183 individuals), was estimated using FSTAT software (Goudet 2002). Both inbreeding and null alleles can cause an excess of homozygotes within a population. Therefore, to avoid confusion and increase estimation accuracy, we jointly obtained unbiased multilocus inbreeding coefficients within the population, frequency of null alleles, and presence of genotyping errors using INEST 2.0 (Chybicki and Burczyk 2009). We applied the individual inbreeding model (Bayesian approach) which can better account for the effect of those factors than the population inbreeding model (maximum likelihood approach), especially in small samples. We performed five models (nfb, nb, nf, fb, n) considering different combinations of three parameters: n (null alleles), f (inbreeding coefficient), and b (genotyping errors), with Markov chain Monte Carlo of 200,000 cycles, burn‐in period of 20,000, and thinning of 1000. Then, by comparing the deviance information criterion (DIC) obtained for each model, we determined which one better fit the data.

### 2.5 Fine‐Scale Spatial Genetic Structure: Indirect Estimate of Gene Flow

Fine‐scale spatial genetic structure was assessed for mature trees (adult cohort) and regeneration (seedling cohort) following the procedure described in Vekemans and Hardy (2004). Pairwise kinship coefficients (*F*_*ij*_) were calculated between individuals using SPAGeDi 1.5d software (Hardy and Vekemans 2002), utilizing both Ritland's and Nason's estimators (Loiselle et al. 1995; Ritland 1996). Ritland's estimator is more powerful in detecting genetic structure, but suffers from a downward bias in the presence of low‐frequency alleles, whereas Nason's estimator has no bias. Ten distance classes for use in SPAGeDi were determined using GenAlEx (Peakall and Smouse 2012), which seeks to encompass a roughly constant number of pairwise comparisons within each distance class (Hardy and Vekemans 2002). Seedling and adult cohorts were analyzed separately, resulting in slightly different distance classes for each. Statistical significance of mean *F*_*ij*_ and *b* values was tested in each distance class using 20,000 permutations. The standard error was estimated using jackknife resampling between loci to calculate 95% confidence intervals around mean *F*_*ij*_ values. To measure the intensity of fine‐scale spatial genetic structure, the *Sp* statistic (Vekemans and Hardy 2004) was calculated by *Sp* = −*b*/(1‐*F*_*1*_), where *b* is the regression slope of the kinship estimator *F*_*ij*_ computed among all pairs of individuals against their geographical distances, and *F*_*1*_ is the mean kinship coefficient among individuals of the first distance class (< 14 m). The standard error of *b* was calculated using jackknife resampling between loci (Hardy et al. 2006). To evaluate the strength of spatial genetic structure between generation cohorts, a heterogeneity test based on Smouse et al. (2008) was run. The departure of the entire correlogram from the null hypothesis of no spatial structure was evaluated employing single‐distance (*t* ^2^) and multi‐distance class criteria (ω). The significance of each criterion was determined on the basis of 1000 permutations using GenAlEx (Peakall and Smouse 2012).

Assuming drift–dispersal equilibrium, we estimated *σ*_*g*_, a gene dispersal distance (*σ* ^*2*^_*g*_ is half the mean‐squared parent–offspring distance), and the neighborhood size, *Nb* = 4*πd*_*e*_ *σ* ^*2*^_*g*_ where *d*_*e*_ is an effective population density of reproductive individuals, using SPAGeDi (Vekemans and Hardy 2004). We used an iterative procedure based on the restricted regression slope (*b*_*r*_) of *F*_*ij*_ on ln (*d*_*ij*_) within a limited distance range *σ*_*g*_ < *d*_*ij*_ < 20 σ_g_, and the theoretical expectation that *Nb* = −(1–*F*_*1*_ *)/b*_*r*_ under isolation by distance. The effective population density, *d*_*e*_, should be a fraction of *d*_*obs*_ (Hardy et al. 2006). The observed density (*d*_*obs*_) was 0.0376 trees/m^2^. As a first approximation, D is the product of the census density and the Ne/N ratio (effective over census population sizes), which typically ranges from 0.5 to 0.1 in natural plant populations. Given that population density may affect fine‐scale genetic structure (e.g., Duminil et al. 2016; Hardy 2009; Sagnard et al. 2011), estimators of *Nb* and *σ*_*g*_ were conducted using various effective densities, including *d*_*obs*_, *d*_*obs*_/2, *d*_*obs*_/3. In addition, to account for potential changes in reproductive success variance over the generations, higher effective densities were also considered (e.g., *d*_*obs*_ * 1.5 and *d*_*obs*_ * 2). For each *d*_*e*_, lower and upper bounds for the 95% confidence interval (CI) of *Nb* were computed as (*F*_*1*_–1)/(*b*_*r*_ + 2SE*b*) and (*F*_*1*_–1)/(*br*—2SE*b*), respectively, SE*b* being the standard error of the *b*_*r*_ estimates obtained by jackknifing over loci (when *b*_*r*_ < 2Se*b*, the upper bound was reported as infinite, ∞). The 95% CI of *σ*_*g*_ was obtained similarly as √*Nb*/(4π*d*_*e*_) using the upper and lower *Nb* bounds (Hardy et al. 2006).

### 2.6 Estimates of Direct Gene Flow: Neighborhood Models

The classical neighborhood model (CNM) (Burczyk et al. 2006) was used to estimate current levels of gene flow using the NMπ 2.0 software (Chybicki 2023). This model estimates the probability of the genealogy of a sample of offspring given their genotypes and spatial positions, together with the information about candidate parents (i.e., genotypes, spatial positions and, if available, phenotypes) (Oddou‐Muratorio et al. 2005; Burczyk et al. 2006). Seedling and adult spatial positions, along with parent‐offspring relationship predictions, were used to calculate pollen and seed dispersal distances. Seed dispersal distances were calculated as the Euclidean distance between a seedling and its seed donor tree. The gender of the putative parents (“femaleness”) was set to 0.5, meaning equal probability of being a seed donor or pollen donor since *N. pumilio* is monoecious. Similarly, pollen dispersal distances were calculated based on the distance between the two predicted parents of a given seedling. The seed migration rate (*m*_*s*_), pollen migration rate (*m*_*p*_), selfing rate (*s*), average seed dispersal distance (*δ*_*s*_), average pollen dispersal distance (*δ*_*p*_), shape of seed dispersal kernel (*b*_*s*_), shape of pollen dispersal kernel (*b*_*p*_) and anisotropy parameters (*k*_*s*_, *k*_*p*_, *a*_*a*_ and *a*_*p*_) were estimated following a step‐wise approach. We also tested whether the diameter at breast height (DBH) and height (HT) of candidate parents affected the probability of being a true seed donor (γ_DBH_, γ_HT_) or pollen donor (β_DBH_, β_HT_) using these variables as selection gradients. Genotyping errors for each locus were provided with the sequencing and loaded into NMπ 2.0. Dispersal was modeled through an exponential‐power kernel. Each model was compared to the best fitting standard model using the Akaike Information Criterion (Akaike 1973), which for a k‐parameter model is defined as AIC = 2 (k‐Ln(L)) (i.e., the smaller AIC, the better model fitting).

The recently developed hierarchical neighborhood model (HNM) (Chybicki et al. 2021), which accounts for the effect of overdispersal (i.e., false discovery rate of reproductive potential due to nonsignificant determinants) and has higher specificity than CNM, was also run in this study. Under this model, parameters are estimated by a Bayesian approach based on the Monte Carlo Markov Chain (MCMC) procedure, by combining several algorithms (Klein et al. 2008; Chybicki et al. 2021) and are adjusted to the case of dispersed offspring (i.e., two unknown parents). The rationale behind the estimations is to be able to select phenotypic variables that might be important for seed donor and/or pollen donor fecundity; therefore, it is relevant to assess the fraction of variance in fecundity (R2) explained by selected variables (Chybicki et al. 2021).

### 2.7 Comparisons of Direct and Indirect Estimates of Gene Dispersal

In a two‐dimensional space, the following relationship applies: *σ* ^*2*^_*g*_ = *σ* ^*2*^_*s*_ *+ 1/2σ* ^*2*^_*p*_ (Crawford 1984), where *σ*_*s*_ and *σ*_*p*_ refer to effective seed and pollen dispersal, respectively. The parameters *δ*_*s*_ *and δ*_*p*_, from the direct method, were converted to *σ*_*s*_ and *σ*_*p*_ according to Oddou‐Muratorio & Klein et al. (2008). Using this equation, we obtained an estimate of gene dispersal from direct estimates of seed and pollen dispersal (neighborhood model) to be compared with the indirect gene dispersal estimate (fine scale spatial genetic structure), which is assumed to account for historical dispersal (Oddou‐Muratorio and Klein 2008).

## 3 Results

### 3.1 Genetic Diversity

After data cleaning, where individuals with at least 50% missing data were removed, a final database comprising 192 adults and 372 seedlings was created and used for downstream analysis (CONICET Repository access: https://ri.conicet.gov.ar/handle/11336/235405). Parentage assignment methods are highly sensitive to the presence of null alleles, genotyping errors, and inbreeding (Huang et al. 2018). Null alleles can result in genotyping errors, leading to the erroneous exclusion of true parents. Elevated levels of inbreeding reduce genetic diversity, complicating the detection and differentiation of potential parents. Addressing these three fundamental aspects together minimizes the likelihood of incorrect parentage assignments (Chybicki and Burczyk 2009; Huang et al. 2018). In our analysis, the model that includes the presence of null alleles and genotyping error effects (the ‘nb’ model) within the population was supported by the lowest DIC value in INEST. The estimated unbiased inbreeding coefficient did not significantly differ from zero (F_IS_ = 0). Two loci showed significant null allele frequency, nNF_040 (0.1182 ± 0.0203) and nNF_052 (0.1101 ± 0.0228), and were subsequently removed from the analysis. The probability of excluding a putative parent pair using the remaining 15 SSR loci reached 100% (estimated with GenAlEx) and therefore all were retained. Eight chloroplast haplotypes were identified, but two were prevalent among most adult trees. Unfortunately, cpSSR showed very low variation and lack of discriminant power, meaning they could not serve the intended purpose of identifying or confirming the seed donor parent, and were discarded. Genetic diversity was similar in both the seedling (H_E_ = 0.705) and the adult generation (0.706), and the same tendency was found for the effective number of alleles (A_E_ = 4.258 (seedling); 4.313 (adult)) and allelic richness (A_R_ = 14.0 (seedling); 14.4 (adult)) (Table 1). Private alleles, by contrast, differed slightly and were higher in the seedling cohort (Ap = 3.667) than adults (Ap = 1.867), although it should be noted that the number of sampled seedlings was twice that of adults.

**TABLE 1:** Genetic diversity parameters for adult and seedling generations of *Nothofagus pumilio*, based on genetic variation from 15 microsatellites. Parameters are: Number of alleles (Na), effective number of alleles (Ae), private alleles (Ap), allelic richness (A_R_), observed and expected heterozygosity (Ho and He), and inbreeding coefficient (F). Standard deviation is shown in parentheses.

	N	Na	Ae	Ap	A_R_	Ho	He	F
Adults	192	14.533 (1.759)	4.313 (0.543)	1.867 (0.496)	14.4 (6.7)	0.733 (0.044)	0.706 (0.038)	−0.040 (0.044)
Seedlings	372	16.333 (1.542)	4.258 (0.523)	3.667 (0.566)	14.0 (5.1)	0.720 (0.042)	0.705 (0.037)	−0.024 (0.045)

### 3.2 Fine‐Scale Spatial Genetic Structure Significant Only at Small Spatial Scales

**FIGURE 2:**
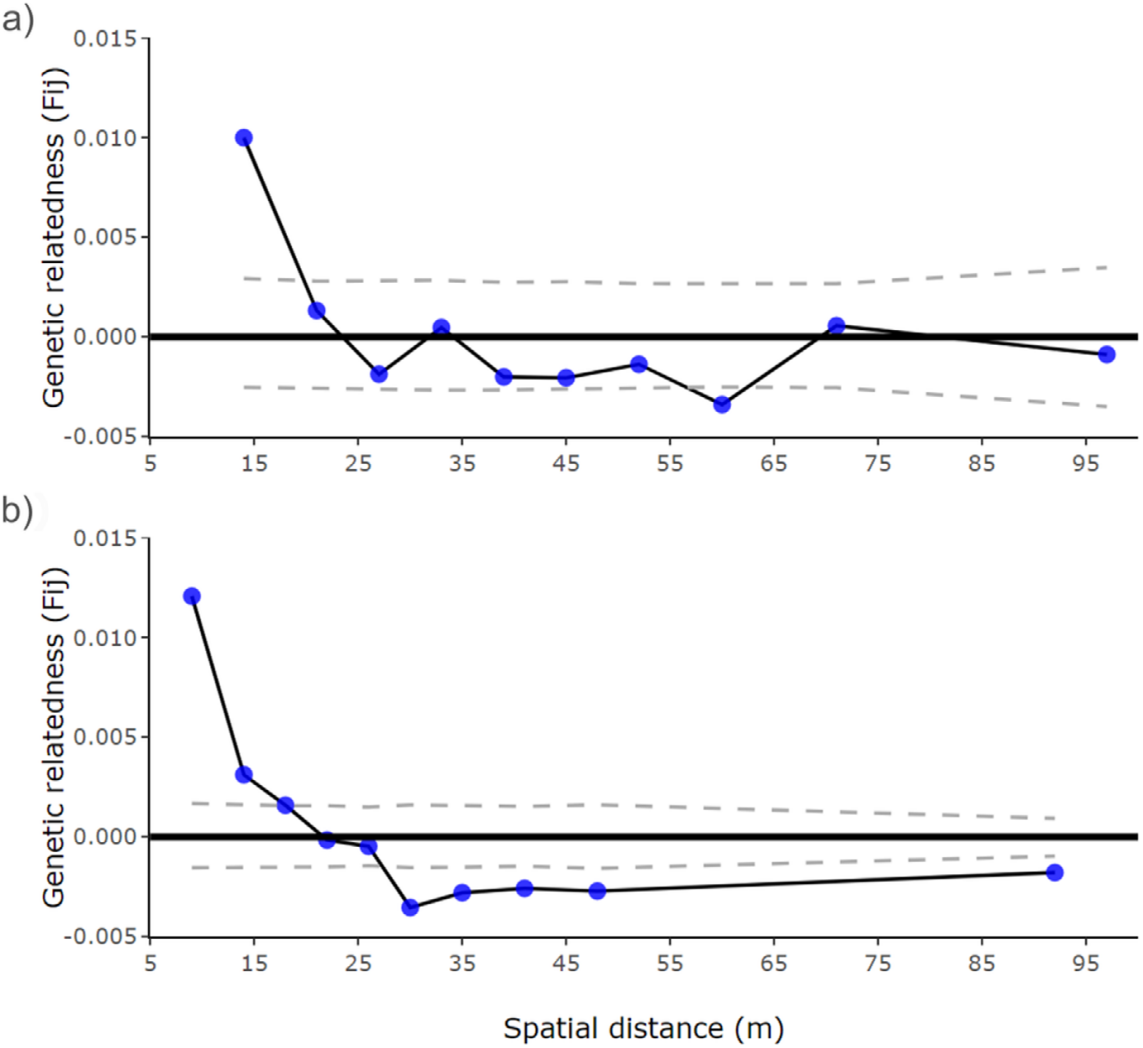
Fine scale Spatial Genetic Structure (FSGS) among the (a) adult trees and (b) seedlings of *Nothofagus pumilio*. Ten distance classes were defined. Dashed lines indicate the 95% confidence intervals.

A significant linear decrease of *Fij*‐coefficient with the logarithm of spatial distance was detected (*p* = 0.0001; Figure 2). Fine‐scale spatial genetic structure was significant (meaning individuals are more related than expected by chance) only at small spatial scales, up to the first distance class in mature trees and the second distance class in seedlings (both 14 m). The intensity of the fine‐scale spatial genetic structure was low (Sp_Ad_ = 0.0049; Sp_Sd_ = 0.0053). Estimates of σ_g_ and N_b_ only converged for values of *d*_*e*_>*d*_*obs*_ (σ_g_ = 33.06; N_b_ = 774). Heterogeneity results were not significantly different between generations at single‐distance class criteria (*t* ^2^) nor multi‐distance class criteria (ω) (*p* > 0.05) (Figure S2).

### 3.3 Estimates of Effective Pollen and Seed Dispersal

The parameters of the neighborhood model estimated using both classical and hierarchical approaches demonstrated high consistency (Table 2, S1), with the hierarchical approach providing a better fit. According to the AIC criterion, the best model incorporates seed and pollen immigration rates (*m*_*s*_ and *m*_*p*_), selfing (*s*), seed and pollen dispersal (*δ*_*s*_ and *δ*_*p*_), the shape of seed and pollen dispersal functions (*b*_*s*_ and *b*_*p*_), seed and pollen anisotropy (*k*_*s*_ and *k*_*p*_), and the direction of seed anisotropy (*a*_*s*_), along with selection gradients for the phenotypic traits (γ_DBH_, γ_HT_, β_DBH_, β_HT_). Seed and pollen immigration rates were estimated as 0.164 and 0.80, respectively. Genotyping errors were low (range 0–0.02). Selfing was not significant, consistent with the INEST results, which estimated s = 0.002. Short average seed and pollen dispersal distances were estimated: δ_s_ = 13.33 m and δ_p_ = 24.08 m (Table 2). However, the shape parameters of the dispersal kernel suggested a fat‐tailed distribution, particularly for the pollen (*b*_*p*_ = 0.31, while seeds were *b*_*s*_ = 0.92), indicating the occurrence of longer‐distance dispersal events, which is consistent with the observed immigration rates.

**TABLE 2:** Estimated parameters obtained with the hierarchical neighborhood model in the studied *N. pumilio* plot. The posterior estimate is listed, including the posterior median with limits of the 95% credible interval (HPDL is lower‐limit and HPDH is upper‐limit).

Parameter	Description	Estimate	HPDL [95%]	HPDH [95%]
m_s_	Seed immigration rate	0.164	0.124	0.206
*s*	Self‐fertilization rate	0.002	0.000	0.009
δ_s_	Mean distance of seed dispersal (m)	13.33	11.70	15.20
b_s_	Shape of exponential power seed dispersal kernel	0.917	0.683	1.17
k_s_	Seed anisotropy	0.494	0.259	0.741
*σ*_***f***_	dispersal of seed donor fecundity around the regression model	0.127	0.985	1.57
*γ*_DBH_	Effect of diameter (DBH) on seed donor fecundity	0.0374	0.0192	0.0556
*γ*_HT_	Effect of height (H_T_) on seed donor fecundity	0	—	—
R^2^_***f***_	Percent of variance in seed donor fecundity explained with the regression model	0.156	0.0323	0.283
m_p_	Pollen immigration rate	0.80	0.749	0.841
δ_p_	Mean distance of pollen dispersal (m)	24.08	20.30	21.90
b_p_	Shape of exponential power pollen dispersal kernel	0.312	0.025	0.786
k_p_	Pollen anisotropy	0.357	0.00002	0.741
*σ*_***m***_	Dispersal of pollen donor fecundity around the regression model	1.29	0.580	2.170
*β*_DBH_	Effect of diameter (DBH) on pollen donor fecundity	0	—	—
*β*_HT_	Effect of height (H_T_) on pollen donor fecundity	0	—	—
R^2^_***m***_	Percent of variance in pollen donor fecundity explained with the regression model	0	—	—

**FIGURE 3:**
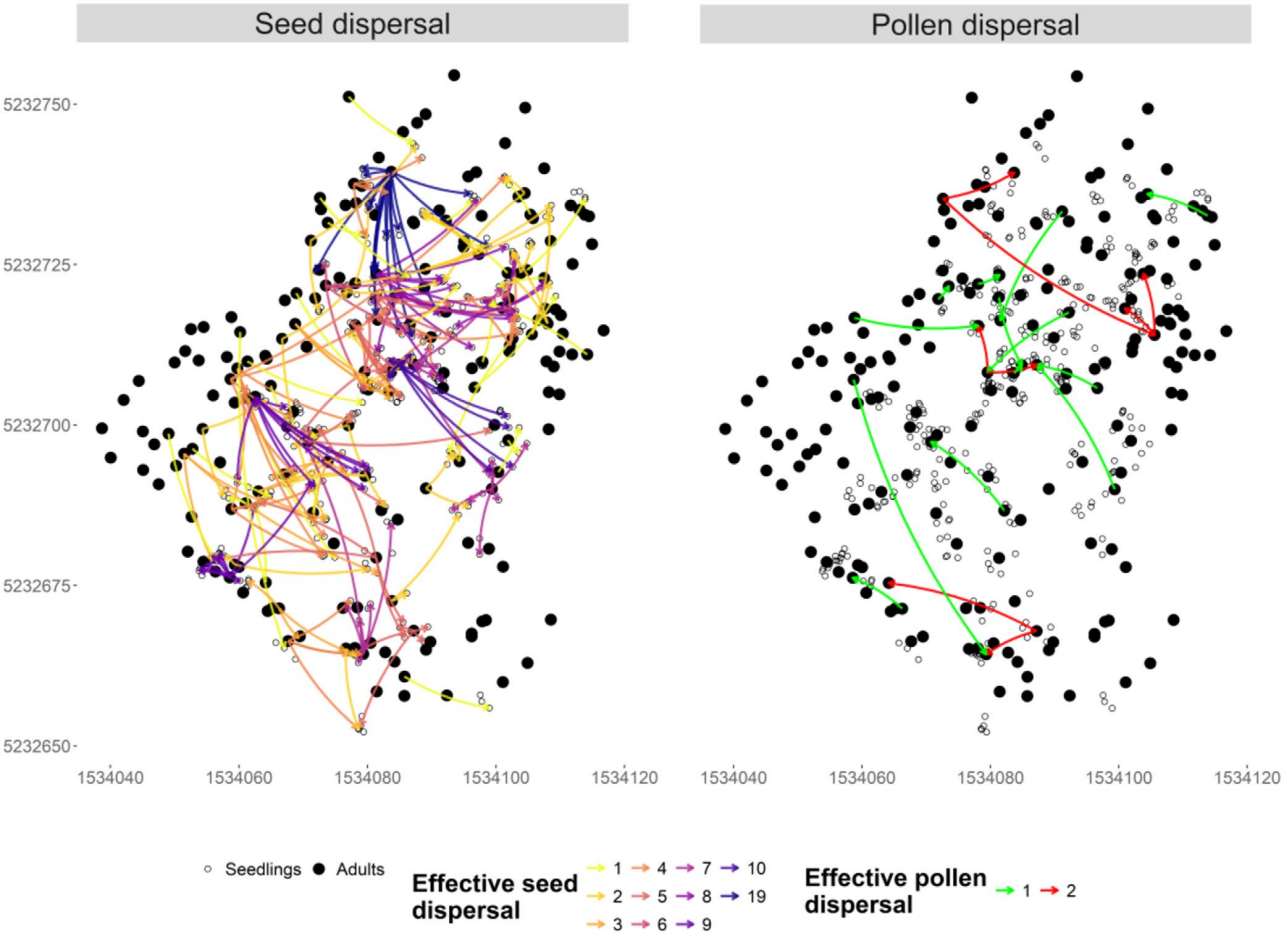
Spatial mapping of the effective seed and pollen dispersal of each adult tree, reconstructed from NMπ runs. Seed dispersal is represented as arrows connecting seed donor trees to all its seedlings with a color gradient from light to dark, indicating the relative number of seedlings produced by each seed donor tree (left panel). Pollen dispersal is represented as arrows connecting the pollen donor adult to the fertilized seed donor adult (right panel).

**FIGURE 4:**
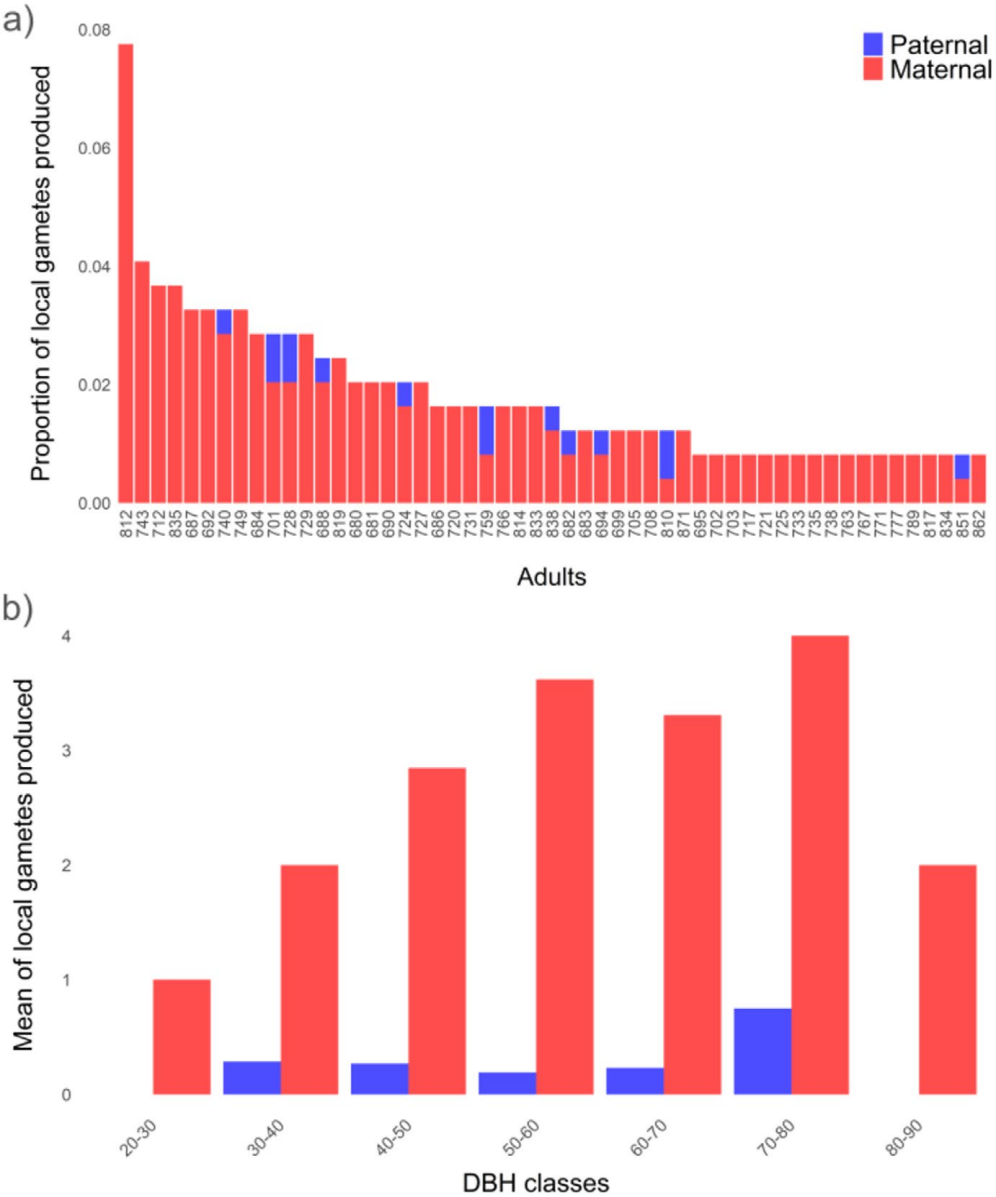
Reproductive success of adult trees by gamete contribution and diameter at breast height (DBH) class; (a) Distributions of individual reproductive success measured as the number of assigned gametes per adult tree. Red bars indicate reproductive success of each tree as seed donor parent, blue bars indicate reproductive success as pollen donor parent. Only adults that contributed with more than 1 gamete were represented; (b) Average number of assigned gametes to adult trees, categorized by DBH class (in cm), showing the distribution of reproductive success among identified parent trees. Red indicates reproductive success of trees as seed donor parents (maternal), blue indicates reproductive success as pollen donor parents (paternal).

Of the 372 seedlings, 84.4% (*n* = 314) were successfully assigned to a local seed donor within the plot (Table S2), indicating localized seed dispersal within the small 0.7 ha plot. Only 16.9% (*n* = 63) could be assigned to both parents, and for the remaining seedlings (15.6%, *n* = 58), no parent could be identified within the stand (Table S2). These results indicate some gamete immigration from external locations. Out of the 192 genotyped adult trees, a total of 83 (43.2%) were identified as parents of 314 seedlings. Spatial mapping of the effective seed and pollen dispersal per adult tree, defined as the number of new seedlings produced by the dispersal activities of a breeding adult, showed that the azimuthal directions of seed and pollen dispersal are heterogeneous (Figure 3). The reproductive success of individual trees, quantified as the number of gametes produced by each adult tree, had a mean value of 1.4 gametes (range 0–19 gametes). The distribution of individual reproductive success was slightly skewed, with 57.8% of the trees with assigned offspring contributing just 1–2 gametes (Figure S3). Meanwhile, a larger proportion of the gametes with at least one identified parent were produced by only 28% of the adult trees (Figure 4a).

Individual posterior median estimates of log‐fecundity ranged from 0.455 to 5.86 for seed donors and from −0.125 to 2.54 for pollen donors (Figure S4). According to the posterior model distribution, the best regression models had probabilities of 0.7614 for seed donor fecundity and 0.7094 for pollen donor fecundity determinants. Through the HNM approach, it was confirmed that diameter (DBH) was significantly associated with seed donor fecundity. Conversely, the same approach excluded the contribution of height for seed donor fecundity, and both DBH and height for pollen donor fecundity. The reproductive success of adult trees along different DBH classes is shown in Figure 4b. The indirect estimate of gene dispersal distance obtained from seed (*δ*_*s*_) and pollen (*δ*_*p*_) dispersal resulted in a value of *σ* ^*2*^_*g*_ = 21.62. This estimate closely aligns with the estimations derived by the hierarchical model.

## 4 Discussion

### 4.1 Gene Dispersal, Reproductive Success, and Fine Scale Spatial Genetic Structure

Our gene flow analysis results suggest that dispersal capacity in the wind‐dispersed *Nothofagus pumilio* is predominantly limited to short distances, with some evidence for longer‐distance dispersal, particularly for pollen. The mean effective dispersal distances for pollen and seeds in *N. pumilio* (*δ*_*p = *_24.08; *δ*_*m = *_13.33) are broadly consistent with those reported in other South American *Nothofagus* species (*δ*_*p*_ < 35 m, Marchelli et al. 2012; *δ*_*p,s*_ < 45 m, Sola et al. 2020). We successfully identified seed donor trees for the majority of analyzed seedlings (> 80%), which aligns with prior seed trap data that showed overall limited seed dispersal, where seeds mainly fell underneath the donor tree's crown (e.g., 20 m from the trunk; Rusch 1993; Cuevas 2000). Meanwhile, the observed contribution of pollen donor trees among the sampled individuals was relatively low (~17% of seedlings), suggesting that most pollen originated outside the sampled plot. These observations align with our first prediction. A caveat to the present study is that our sampled site was quite small (0.7 ha) and the maximum possible distance for detecting pollen dispersal was short (100 m), although this limitation does not necessarily preclude useful conclusions. For example, a similar analysis from a wind‐pollinated species from the same Order, *Fagus sylvatica* , indicated that most mating occurs between adjacent trees (< 20 m), with a few longer‐distance pollination events observed (1–4 km) (Ouayjan and Hampe 2018). Although the exact distances of foreign pollen dispersal cannot be quantified here, rare longer‐distance pollination events should be considered a probable factor in the species' dispersal strategy. Supporting evidence comes from immigration rates, which are significantly different from zero for both seeds and pollen. This aligns with the results reported in other species with similar ecological requirements and dispersal capabilities, namely wind pollination (Oddou‐Muratorio et al. 2011; Leonarduzzi et al. 2016; Semizer‐Cuming et al. 2021). Similarly, fat‐tailed dispersal curves have been reported for several other wind‐dispersed tree species, indicating the relevance of distant dispersal as an autoecological strategy (Robledo‐Arnuncio and Gil 2005; Kremer et al. 2012; Piotti et al. 2012; Gerber et al. 2014; Angbonda et al. 2021).

The overall reproductive success of individual adult trees had an L‐shaped distribution, meaning that a few trees contributed to many seedlings, and one seed donor tree was particularly successful (*n* = 22 seedlings). Such uneven contributions are commonly observed in trees (e.g., Chybicki and Burczyk 2013), and they can have a strong impact on effective population size that would affect the response to selection of adaptive traits (Gerber et al. 2014). An important note is that the individual trees contributing high amounts of seedlings probably differ across years due to the species' masting strategy. A multi‐year study would be needed to understand the long‐term impact of seed loading and masting events on genetic diversity and adaptation.

Consistent with the short seed dispersal distances observed, which are likely influenced by gravity (seeds are wingless and relatively heavy), fine‐scale genetic structure was detected in both adult and seedling cohorts in the first distance class. The strength of the fine‐scale spatial genetic structure, as inferred by the *Sp* statistic (*Sp*_Ad_ = 0.0049; *Sp*_Sd_ = 0.0053), is comparable to previous reports for *N. pumilio* (Mathiasen and Premoli 2013, *Sp* = 0.003–0.006; Soliani et al. 2016, *Sp* = 0.001–0.012), other *Nothofagus* species (Sola et al. 2022, *Sp* = 0.0089–0.0161) and similar to values found in related *Fagus* species (e.g., Sjölund and Jump 2015, *Sp* = 0.0032–0.0114). However, this fine‐scale genetic structure does not appear to promote interbreeding between relatives, as the inbreeding coefficient was below zero. Selfing in this and other *Nothofagus* species is generally negligible (Sola et al. 2020), which has been attributed to prezygotic barriers on the stigmatic surface (Torres and Puntieri 2013). Moreover, levels of genetic diversity observed in the seedlings suggest that there is no loss of genetic variation from one generation to the next. Overall, this suggests that fine‐scale spatial genetic structure and short seed dispersal may not be detrimental to the species.

Our findings suggest that the most mature and larger seed donor trees (i.e., those with greater DBH) had greater contributions to reproduction. In theory, larger individuals have more branches and reproductive organs and, therefore, increased fecundity (Enquist and Niklas 2002). This relationship between adult DBH and reproductive success has been observed in numerous tree species. In some cases, it is associated with the seed donor tree (e.g., Sola et al. 2020; Asuka et al. 2005; Oddou‐Muratorio et al. 2010), as we found in *N. pumilio* . In other cases, it may be linked to the pollen donor tree (e.g., Angbonda et al. 2021; Monthe et al. 2021); however, our results did not support this. Therefore, our prediction was only validated for the seed donors. It is possible that the low number of seedlings assigned to a pollen donor reduced study power to detect such an association. The reproductive success of adults can directly influence the establishment and subsequent development of young plants in the understory at the microsite level, as was reported in *Nothofagus* species (e.g., Soler Esteban et al. 2013; Bahamonde et al. 2018). However, *N. pumilio* regeneration success can also be affected by environmental factors, including variation in soil moisture, light availability, canopy density, seed predation, and even wind as the primary pollen dispersal mechanism (Martínez Pastur et al. 2011). For example, these environmental influences are reflected in fluctuations in flowering, seeding, and seedling recruitment patterns (Martínez Pastur et al. 2011, 2013). Thus, individual adults' reproductive success and dispersal distances are just a few of the factors affecting *N. pumilio* regeneration, and further study regarding environmental factors could be considered.

The historical dispersal capacity estimated for this tree species, as inferred from indirect methods, is comparable to contemporary dispersal capacity. This suggests minimal human intervention in the stand, especially in combination with the uneven‐aged structure of the forest, which contains standing trees of various diameters (see supplement) interspersed with regeneration from masting pulses, reflecting the typical gap dynamics characteristic of this light‐demanding species. Historical dispersal distances also have implications for past demographic processes. In species that have evolved and adapted to a specific environment or particular environmental conditions, like the cold‐tolerant *Nothofagus pumilio*, their limited dispersal aligns with a low capacity to compete with other species for colonizing new sites (e.g., Thompson and Fronhofer 2019). This could affect their ability to track environmental change (e.g., Torres et al. 2024). There is a need to consider ecological and evolutionary processes together to understand the trajectory of adapted species that primarily persist within their current ranges, do not exhibit range shifts, and show limited dispersal capacity. As a nonpioneer species, *N. pumilio* is usually found in mature forests, and its irregular seed production and variable seed quality further hinder its potential range shifts. The recalcitrant condition of their seeds in nature, combined with these factors, limits its dispersal ability (Soliani et al. 2021).

### 4.2 Dispersal Capacity Related With Conservation of Genetic Resources and Strategies Facing Climate Change

Gene flow capabilities are critical under the threat of climate change since gene flow directly affects migration rates and has the power to strengthen or weaken local adaptation patterns (Tigano and Friesen 2016). A recent landscape genomics study of *N. pumilio* found multiple gene regions under selection that were strongly associated with environmental clines related to temperature and precipitation (Sekely et al. 2024). Transplant experiments have similarly found evidence of genetic adaptation along environmental clines (Mathiasen and Premoli 2016), albeit with some evidence of phenotypic plasticity. Since local adaptation is present despite gene flow, it suggests that local selection pressures are strong (Kawecki and Ebert 2004). However, climate change is likely to alter these selection pressures. Weather station data adjacent to our sampling site has already recorded noticeable shifts in recent climate trends. For instance, in 2016, the annual accumulated precipitation was lower and the average annual temperature was higher than usual, indicating a dry year, while in 2017, absolute minimum temperatures were recorded well below the average values. Such temperature shifts could also strongly impact wind patterns, which could have direct consequences for the species' dispersal and gene flow patterns. Shifting trends in key climate variables could also impact the species during the early stages of the regeneration cycle.

To avoid the extinction of local gene pools, populations will need to either migrate or adapt from one generation to the next (Aitken et al. 2008). For migration, the future suitability of both existing habitats and potential new habitats is an important factor, in addition to dispersal capacity. A recent modeling study of future *N. pumilio* distribution, based on the perspectives of climate change by 2070, showed a significant loss of suitable habitat within its current distribution area (~40%) (Soliani et al. 2024). Predicted loss areas are mainly associated with the edges, i.e., ecotonal forest –steppe areas, and the northern limits of its current distribution, where the effect of climate change may be more drastic and where some of the more genetically diverse populations currently exist (Sekely et al. 2024). However, niche modeling has the drawback of not considering crucial life history traits that can impact distribution, such as dispersal (Kearney and Porter 2009). The limited dispersal ability detected by our results could exacerbate the problem of suitable habitat loss, since potential colonization areas are reduced. For example, *N. pumilio* is a treeline species with somewhat limited potential for altitudinal range shift. Rising temperatures are expected to push global treeline ecotones upslope, but the interaction between temperature increase and variations in precipitation has been shown to affect the ability of *N. pumilio* to regenerate and thus actually migrate upslope (Srur et al. 2018). Since migration capacity is uncertain, adaptation in situ is another important possibility. Previous studies have reported that northern populations in particular contain greater amounts of overall diversity (Soliani et al. 2015; Mattera et al. 2020; Sekely et al. 2024), suggesting they could have greater adaptive capacity. Our results indicate that neutral genetic diversity is high in the studied plot, and notably, that it is stable across generations.

The rate of contemporary climate change throws the future of this species into further uncertainty. Knowledge about dispersal patterns plays a crucial role in genetic resource conservation and the development of future strategies to mitigate possible impacts of climate change. Our results suggest limited seed dispersal capacity of *N. pumilio*, meaning there is a low probability of natural expansion beyond the current distributional range, particularly at the timescales required to keep pace with rapid climate change. While we also reported high diversity, adaptation rates may still be too slow in relation to climate change. One possible rescue technique might be human‐assisted gene flow, which involves the movement of seeds within the current distribution range, or migration, where colonization is assisted beyond the current range edges. The general goal of assisted migration is *ex situ* conservation, for which promoting high overall genetic diversity and ensuring effective genetic mixing in successive generations is crucial. A particularly useful tool for implementing an assisted migration program would be the establishment of genetic management units, which can provide guidelines for the movement of seminal material. For example, a prior study using neutral markers identified six high‐diversity conservation areas as potential genetic management units (Mattera et al. 2020), one of which contains the present study's sampling site. Using such units to inform assisted migration could be a potential mitigation strategy to address the species' limited dispersal, although further analysis would be required (e.g., Browne et al. 2019).

## 5 Conclusion

The wind‐dispersed and cold‐adapted Patagonian tree species *Nothofagus pumilio* is expected to be strongly affected by climate change, and it may face additional challenges due to the limited seed dispersal capacity described here. *N. pumilio* plays a crucial ecological role as a foundation species of Patagonian Andes forests, and its decline could lead to both direct and indirect impacts on other accompanying species. Our genetics‐informed seed and pollen dispersal and fine‐scale spatial genetic structure analyses suggested some immigration and longer‐distance dispersal events, particularly for pollen, which could facilitate allele migration but not population migration. In light of these identified gene flow limitations, future conservation efforts may best be focused on *ex situ* conservation, including forest plantations, seed orchards, and possibly assisted migration initiatives.

## Author Contributions

**C. Soliani:** conceptualization (equal), formal analysis (equal), funding acquisition (equal), investigation (equal), methodology (equal), resources (equal), validation (equal), visualization (equal), writing – original draft (lead). **J. Sekely:** conceptualization (equal), formal analysis (equal), investigation (equal), resources (equal), visualization (equal), writing – original draft (lead). **C. Zamora‐Ballesteros:** data curation (equal), formal analysis (equal), visualization (equal), writing – original draft (equal). **K. Heer:** conceptualization (equal), funding acquisition (equal), resources (equal), writing – review and editing (equal). **O. Lepais:** formal analysis (equal), methodology (equal), validation (equal), writing – review and editing (equal). **V. Mondino:** resources (equal), writing – review and editing (equal). **L. Opgenoorth:** conceptualization (equal), funding acquisition (equal), writing – review and editing (equal). **M. Pastorino:** funding acquisition (equal), investigation (equal), resources (equal), writing – review and editing (equal). **P. Marchelli:** conceptualization (equal), data curation (equal), formal analysis (equal), funding acquisition (equal), investigation (equal), resources (equal), supervision (equal), validation (equal), visualization (equal), writing – original draft (lead).

## Conflicts of Interest

The authors declare no conflicts of interest.

## Benefit Sharing Statement

Benefits from this research accrue from the sharing of our data and results on public databases, as described above.

## Supporting information


Data S1.

## Data Availability

Raw sequence reads are deposited in the European Nucleotide Archive (ENA accession number: ERR13472835). Individual genotype data are available on the Repository of the National Research Council of Argentina (CONICET Repository access: https://ri.conicet.gov.ar/handle/11336/235405).
