# Supplementary material for: Restricted Dispersal in the Late Successional Forest Tree Species Nothofagus Pumilio: Consequences Under Global Change
Source: Ecol Evol. 2025 May 23;15(5):e71002. doi: 10.1002/ece3.71002 (PMC12100765; doi:10.1002/ece3.71002)
Supplement: Supplementary file 1 — Data S1. [file ECE3-15-e71002-s001.zip › Supplementary_Soliani-Sekely et al.docx]

Restricted dispersal in the late successional forest tree species *Nothofagus pumilio*: consequences under global change

Soliani C^1,*,#^, Sekely J^2,3,*^, Zamora-Ballesteros C^2^, Heer K^2^, Lepais O^4^, Mondino V^5^, Opgenoorth L^3^ , Pastorino M^1^ and P Marchelli^1^

^1^ INTA Bariloche, Instituto de Investigaciones Forestales y Agropecuarias Bariloche IFAB (INTA-CONICET). Modesta Victoria 4450 (8400) S.C. Bariloche, Argentina

^2^ Eva Mayr Stihl Professorship for Forest Genetics, Albert-Ludwigs Universität Freiburg, Bertoldstraße 17, 79098 Freiburg, Germany

^3^ Plant Ecology and Geobotany, Philipps-Universität Marburg, Karl-von-Frisch-Straße 8, 35032 Marburg, Germany

^4^ Univ. Bordeaux, INRAE, BIOGECO, F-33610, Cestas, France

^5^ INTA Esquel Chacabuco 513 (9200) Esquel, Chubut, Argentina

* These authors share first co-authorship

# Author for correspondence: soliani.carolina@inta.gob.ar

Supplementary material containing Figures S1, S2, S3, S4, S5, and Tables S1, S2

(a)


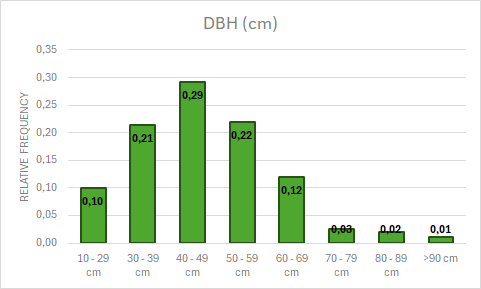


(b)


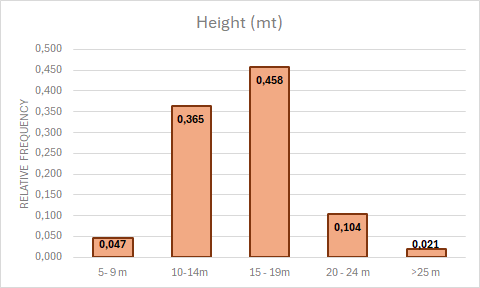


Figure S1: Frequency distribution of diameter (a) and height (b) classes, expressed in centimetres and metres, respectively, among all the adult individuals included in the analysis.


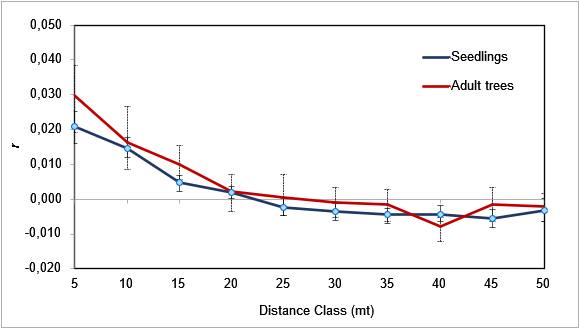


Figure S2: Spatial genetic structure employing single-distance (*t*^2^) and multi-distance class criteria (ω) through a heterogeneity test for both adults and seedling cohorts based on (Smouse et al 2008)


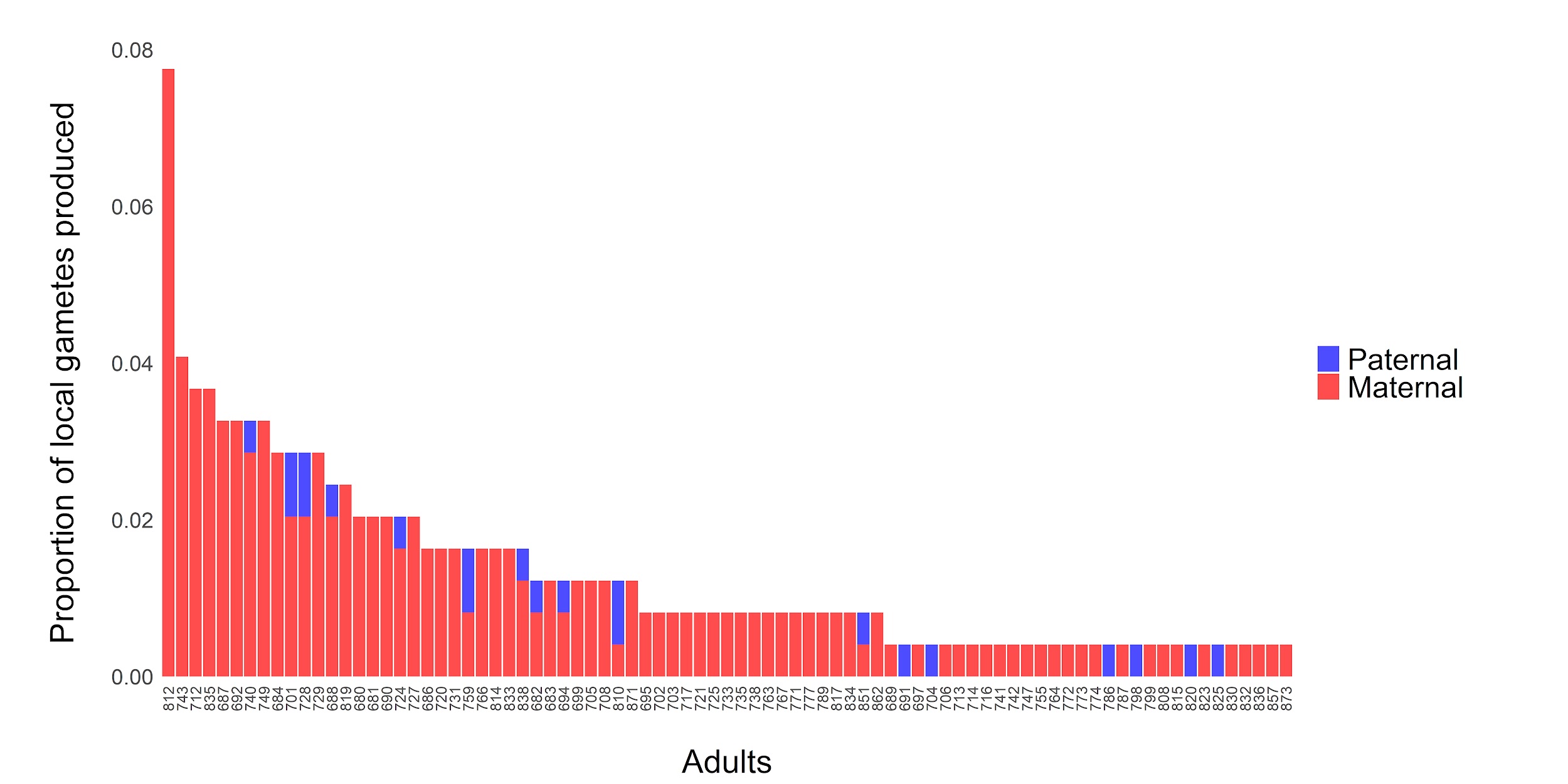


Figure S3: Distributions of individual reproductive success measured as the number of assigned gametes per adult tree. Red bars indicate reproductive success of each tree as female parent, blue bars indicate reproductive success as male parent


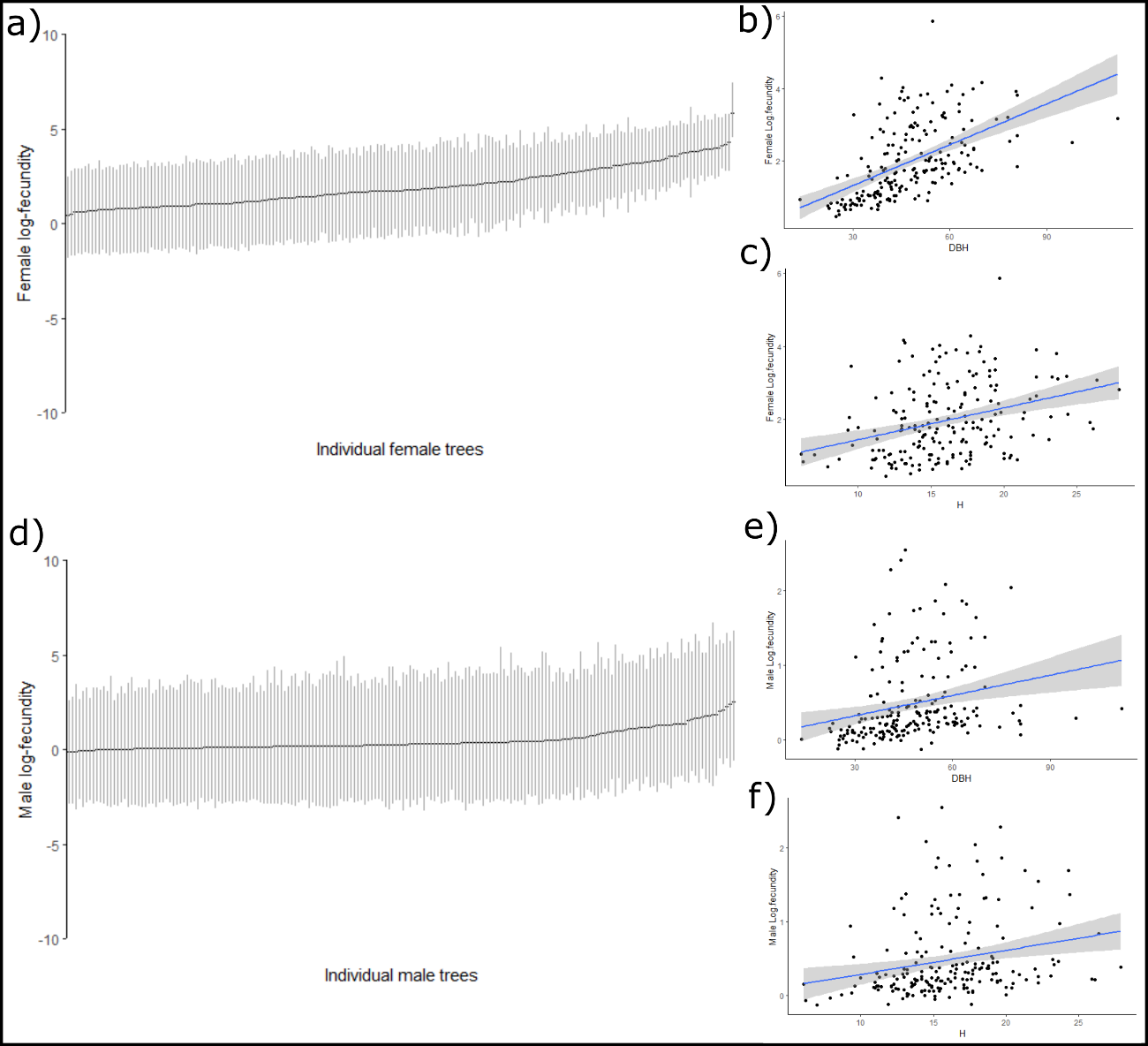


Figure S4: Estimated log-fecundity values for female (a) and male (d) trees in *Nothofagus pumilio* plot. The relationship between log-fecundity and tree phenotypic traits for female (b, c) and male (e, f) parents.


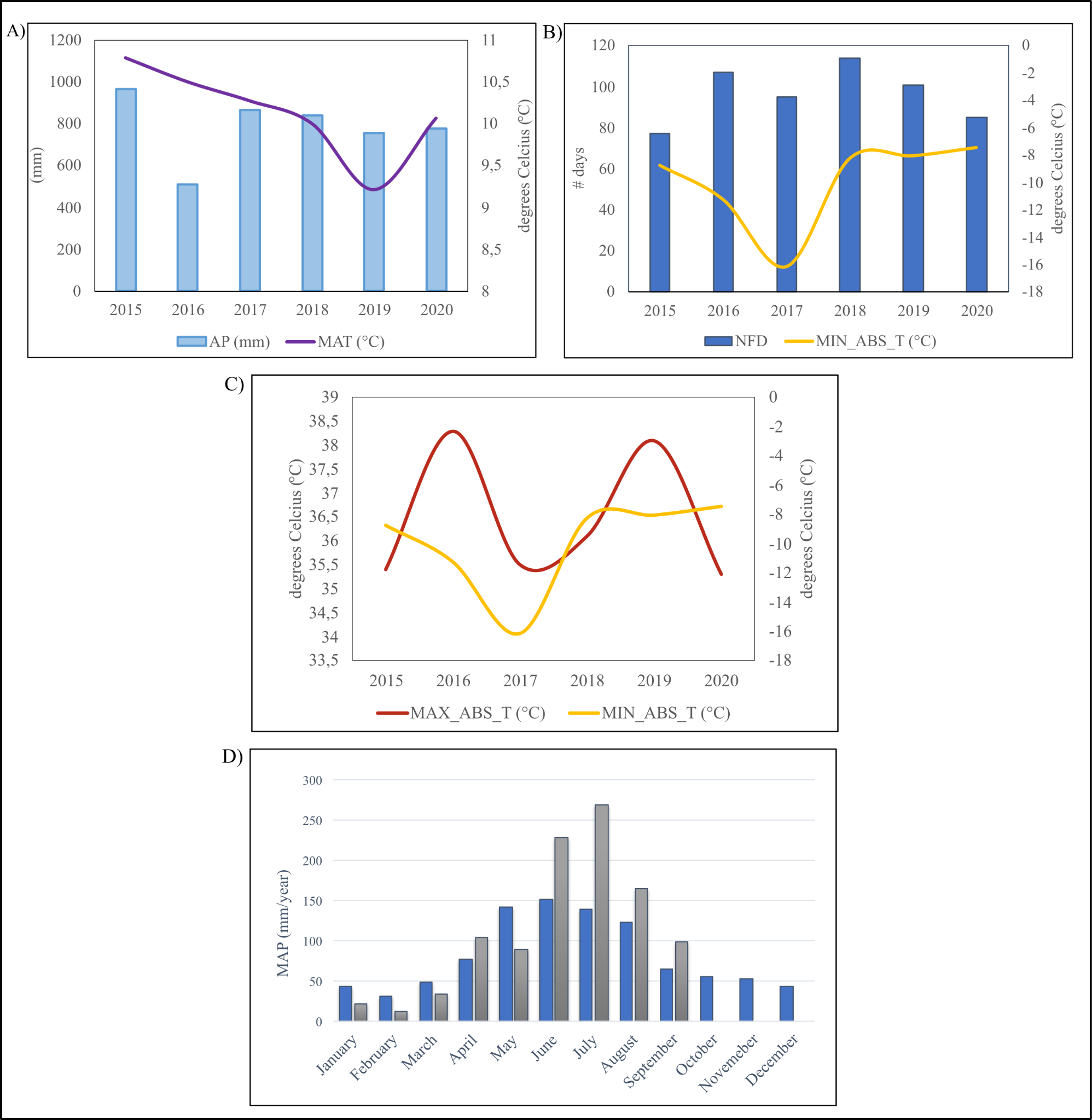


Figure S5: Climatic data from the nearest meteorological station from the studied plot, located at INTA Trevelin (-43.121 S, -71.561 W). A) Accumulated precipitation in mm (AP) in relation to mean annual temperature (MAT) in °C degrees; B) Number of frost days (NFD) in relation to minimum absolute temperatures (MIN_ABS_T) registered in the five previous years from sampling; C) Maximum (MAX_ABS_T) and minimum absolute temperatures (MIN_ABS_T) registered in the five previous years from sampling; d) Historical mean annual precipitation (MAP) of the period 1970-2022 (blue) in relation to the last year 2023 (grey) with unusual/abundant precipitations.

Table S1: Estimated parameters obtained with the classical neighborhood model in the studied *N. pumilio* plot

| Parameter | Description | Estimate (95% CI) |
| --- | --- | --- |
| m_s_ | Seed immigration rate | 0.166 (0.144, 0.188) |
| *s* | Self-fertilization rate | 0.0 (-0.001, 0.001) |
| δ_s_ | Mean distance of seed dispersal (m) | 13.340 (12.607, 14.164) |
| b_s_ | Shape of exponential power seed dispersal kernel | 0.916 (0.803, 1.030) |
| k_s_ | Seed anisotropy | 0.459 (0.365, 0.552) |
| 𝛄_DBH_ | Effect of diameter (DBH) on female fecundity | 0.021 (0.016, 0.025) |
| 𝛄_HT_ | Effect of height (H_T_) on female fecundity | 0.028 (0.018, 0.039) |
| m_p_ | Pollen immigration rate | 0.801 (0.778, 0.825) |
| δ_p_ | Mean distance of pollen dispersal (m) | 16.562 (13.135, 22.406) |
| b_p_ | Shape of exponential power pollen dispersal kernel | 0.623 (0.406, 0.841) |
| k_p_ | Pollen anisotropy | -0.304 (-0.645, 0.037) |
| *β*_DBH_ | Effect of diameter (DBH) on male fecundity | 0.051 (0.032, 0.071) |
| *β*_HT_ | Effect of height (H_T_) on male fecundity | 0.060 (0.013, 0.106) |

Table S2: Inferred genealogies for the 372 seedlings of the studied plot. Adult trees, both mother and fathers, were identified as correlative numbers within the plot, this ID is the same as used in CONICET Repository access: https://ri.conicet.gov.ar/handle/11336/235405 [Most probable genealogies are indicated as Mo1, Fa1 with probability Pr1]

| **Prog** | **Mo1** | **Fa1** | **Pr1** | **Mo2** | **Fa2** | **Pr2** |
| --- | --- | --- | --- | --- | --- | --- |
| **1** | 680 | 701 | 0.8564 | 701 | 680 | 0.1436 |
| **2** | 701 | -1 | 0.9864 | -1 | -1 | 0.0136 |
| **3** | -1 | -1 | 0.9998 | 690 | -1 | 0.0002 |
| **4** | 702 | -1 | 0.9046 | -1 | -1 | 0.0954 |
| **5** | 702 | -1 | 0.9648 | -1 | -1 | 0.0348 |
| **6** | 701 | -1 | 0.9984 | -1 | -1 | 0.0016 |
| **7** | 681 | -1 | 0.994 | 705 | -1 | 0.006 |
| **8** | 703 | -1 | 0.9524 | -1 | -1 | 0.044 |
| **9** | 681 | 704 | 0.9714 | 681 | -1 | 0.019 |
| **10** | -1 | -1 | 0.9558 | 705 | -1 | 0.044 |
| **11** | 680 | 825 | 0.9104 | 825 | 680 | 0.081 |
| **12** | 680 | -1 | 0.9996 | 766 | -1 | 0.0002 |
| **13** | 680 | -1 | 1 | -1 | -1 | 0 |
| **14** | 683 | -1 | 1 | -1 | -1 | 0 |
| **15** | 701 | -1 | 0.942 | 683 | -1 | 0.058 |
| **16** | 683 | 684 | 0.679 | 684 | 683 | 0.3208 |
| **17** | 683 | -1 | 0.9986 | -1 | -1 | 0.0014 |
| **18** | 682 | -1 | 1 | -1 | -1 | 0 |
| **19** | 686 | -1 | 0.9994 | -1 | -1 | 0.0006 |
| **20** | 683 | -1 | 1 | -1 | -1 | 0 |
| **21** | 743 | 750 | 0.531 | 743 | -1 | 0.4682 |
| **22** | 743 | -1 | 1 | -1 | -1 | 0 |
| **23** | 684 | 851 | 0.8318 | 851 | 684 | 0.0898 |
| **24** | -1 | -1 | 0.906 | 701 | -1 | 0.094 |
| **25** | 682 | -1 | 0.9998 | -1 | -1 | 0.0002 |
| **26** | -1 | -1 | 0.93 | 800 | -1 | 0.0618 |
| **27** | 687 | -1 | 0.962 | 744 | -1 | 0.0334 |
| **28** | 686 | -1 | 0.6942 | 687 | -1 | 0.2992 |
| **29** | 684 | 740 | 0.8288 | 740 | 684 | 0.1648 |
| **30** | 701 | 691 | 0.8992 | 691 | 701 | 0.1008 |
| **31** | 690 | -1 | 1 | -1 | -1 | 0 |
| **32** | 686 | 684 | 0.6298 | 684 | 686 | 0.2116 |
| **33** | 684 | -1 | 0.6 | -1 | -1 | 0.3998 |
| **34** | 684 | 701 | 0.8322 | 701 | 684 | 0.1676 |
| **35** | 687 | 686 | 0.6354 | 686 | 687 | 0.3624 |
| **36** | 688 | -1 | 0.5344 | 687 | -1 | 0.4376 |
| **37** | 690 | -1 | 0.9356 | 687 | -1 | 0.0644 |
| **38** | 687 | -1 | 0.999 | 791 | -1 | 0.0006 |
| **39** | 687 | -1 | 0.9994 | -1 | -1 | 0.0006 |
| **40** | 690 | -1 | 0.9998 | 687 | -1 | 0.0002 |
| **41** | 687 | -1 | 1 | -1 | -1 | 0 |
| **42** | 688 | 680 | 0.5338 | 680 | 688 | 0.448 |
| **43** | 690 | -1 | 0.999 | -1 | -1 | 0.001 |
| **44** | 688 | -1 | 0.9786 | -1 | -1 | 0.0196 |
| **45** | -1 | -1 | 0.9676 | 835 | -1 | 0.0304 |
| **46** | 688 | -1 | 1 | -1 | -1 | 0 |
| **47** | 812 | -1 | 0.9978 | -1 | -1 | 0.0014 |
| **48** | 686 | -1 | 0.9134 | 701 | -1 | 0.0866 |
| **49** | 819 | -1 | 1 | -1 | -1 | 0 |
| **50** | 812 | -1 | 0.9936 | 688 | -1 | 0.0064 |
| **51** | 724 | -1 | 1 | -1 | -1 | 0 |
| **52** | 694 | -1 | 0.6534 | 692 | -1 | 0.3462 |
| **53** | 812 | -1 | 0.9992 | -1 | -1 | 0.0008 |
| **54** | 743 | -1 | 0.715 | -1 | -1 | 0.2764 |
| **55** | 701 | -1 | 0.9994 | -1 | -1 | 0.0006 |
| **56** | 692 | -1 | 0.9874 | -1 | -1 | 0.01 |
| **57** | 686 | 766 | 0.4164 | 766 | 686 | 0.2604 |
| **58** | 812 | -1 | 0.9994 | -1 | -1 | 0.0004 |
| **59** | 692 | -1 | 0.9992 | -1 | -1 | 0.0006 |
| **60** | 690 | 798 | 0.8244 | 798 | 690 | 0.1744 |
| **61** | 812 | -1 | 1 | -1 | -1 | 0 |
| **62** | 812 | 810 | 0.8994 | 810 | 812 | 0.1006 |
| **63** | -1 | -1 | 0.5016 | 695 | -1 | 0.4982 |
| **64** | 812 | 695 | 0.6896 | 695 | 812 | 0.308 |
| **65** | 812 | -1 | 1 | -1 | -1 | 0 |
| **66** | 694 | 695 | 0.5218 | 695 | 694 | 0.2998 |
| **67** | 812 | -1 | 0.9998 | -1 | -1 | 0.0002 |
| **68** | 812 | -1 | 1 | -1 | -1 | 0 |
| **69** | 694 | -1 | 1 | -1 | -1 | 0 |
| **70** | 812 | -1 | 0.9998 | 817 | -1 | 0.0002 |
| **71** | -1 | -1 | 0.9978 | 801 | -1 | 0.002 |
| **72** | -1 | -1 | 1 | -1 | -1 | 0 |
| **73** | 695 | -1 | 0.653 | -1 | -1 | 0.3412 |
| **74** | 695 | -1 | 0.899 | -1 | -1 | 0.1002 |
| **75** | 819 | 820 | 0.8518 | 820 | 819 | 0.1478 |
| **76** | 717 | -1 | 1 | -1 | -1 | 0 |
| **77** | 819 | 812 | 0.6472 | 812 | 819 | 0.3528 |
| **78** | -1 | -1 | 0.751 | 746 | -1 | 0.2476 |
| **79** | 819 | -1 | 1 | -1 | -1 | 0 |
| **80** | -1 | -1 | 1 | -1 | -1 | 0 |
| **81** | 812 | -1 | 0.9992 | -1 | -1 | 0.0008 |
| **82** | -1 | -1 | 1 | -1 | -1 | 0 |
| **83** | 819 | 695 | 0.694 | 695 | 819 | 0.1426 |
| **84** | 819 | -1 | 0.9998 | -1 | -1 | 0.0002 |
| **85** | -1 | -1 | 0.9992 | 790 | -1 | 0.0004 |
| **86** | 823 | -1 | 1 | -1 | -1 | 0 |
| **87** | 819 | -1 | 0.953 | -1 | -1 | 0.0456 |
| **88** | 697 | -1 | 0.9494 | 724 | -1 | 0.049 |
| **89** | 714 | 718 | 0.5068 | 718 | 714 | 0.4928 |
| **90** | 699 | -1 | 0.8464 | 699 | 730 | 0.1464 |
| **91** | -1 | -1 | 0.97 | 699 | -1 | 0.03 |
| **92** | 819 | 699 | 0.5778 | 699 | 819 | 0.418 |
| **93** | 699 | -1 | 0.9952 | 705 | 699 | 0.0034 |
| **94** | 680 | -1 | 1 | -1 | -1 | 0 |
| **95** | 702 | 701 | 0.71 | 701 | 702 | 0.2892 |
| **96** | -1 | -1 | 0.7078 | 699 | -1 | 0.2922 |
| **97** | 699 | -1 | 1 | -1 | -1 | 0 |
| **98** | -1 | -1 | 1 | -1 | -1 | 0 |
| **99** | 681 | -1 | 0.9998 | -1 | -1 | 0.0002 |
| **100** | -1 | -1 | 0.9996 | 703 | -1 | 0.0004 |
| **101** | 716 | 714 | 0.542 | 714 | 716 | 0.328 |
| **102** | 720 | -1 | 0.9998 | -1 | -1 | 0.0002 |
| **103** | 705 | 706 | 0.6722 | 706 | 705 | 0.3276 |
| **104** | 729 | -1 | 1 | -1 | -1 | 0 |
| **105** | 681 | -1 | 1 | -1 | -1 | 0 |
| **106** | 703 | -1 | 0.8464 | -1 | -1 | 0.0862 |
| **107** | 708 | -1 | 1 | -1 | -1 | 0 |
| **108** | -1 | -1 | 0.9998 | 696 | -1 | 0.0002 |
| **109** | 708 | -1 | 0.9998 | -1 | -1 | 0.0002 |
| **110** | 705 | -1 | 0.9996 | -1 | -1 | 0.0004 |
| **111** | 706 | 727 | 0.7632 | 727 | 706 | 0.2352 |
| **112** | 717 | -1 | 0.9902 | -1 | -1 | 0.0098 |
| **113** | 708 | -1 | 1 | -1 | -1 | 0 |
| **114** | 705 | -1 | 0.992 | 787 | -1 | 0.0054 |
| **115** | 706 | -1 | 0.9996 | -1 | -1 | 0.0004 |
| **116** | 727 | -1 | 1 | -1 | -1 | 0 |
| **117** | -1 | -1 | 0.7282 | 706 | -1 | 0.241 |
| **118** | 817 | -1 | 0.7406 | 700 | -1 | 0.1796 |
| **119** | 729 | -1 | 0.9992 | -1 | -1 | 0.0008 |
| **120** | 836 | 728 | 0.8342 | 728 | 836 | 0.1654 |
| **121** | 835 | -1 | 0.8956 | -1 | -1 | 0.0898 |
| **122** | 727 | -1 | 0.9586 | 728 | -1 | 0.0294 |
| **123** | -1 | -1 | 1 | -1 | -1 | 0 |
| **124** | 712 | -1 | 0.843 | 712 | 835 | 0.1368 |
| **125** | 724 | -1 | 0.9308 | -1 | -1 | 0.069 |
| **126** | 725 | -1 | 0.9374 | 869 | -1 | 0.0614 |
| **127** | 725 | -1 | 1 | -1 | -1 | 0 |
| **128** | 705 | -1 | 0.9854 | -1 | -1 | 0.0146 |
| **129** | 713 | -1 | 0.9998 | -1 | -1 | 0.0002 |
| **130** | -1 | -1 | 0.9996 | 879 | -1 | 0.0004 |
| **131** | -1 | -1 | 1 | -1 | -1 | 0 |
| **132** | 724 | -1 | 1 | -1 | -1 | 0 |
| **133** | 724 | 717 | 0.6288 | 717 | 724 | 0.3702 |
| **134** | 718 | -1 | 0.6694 | -1 | -1 | 0.3284 |
| **135** | 718 | 714 | 0.5068 | 714 | 718 | 0.4932 |
| **136** | 718 | -1 | 0.51 | -1 | -1 | 0.49 |
| **137** | 710 | -1 | 0.7636 | 879 | -1 | 0.1344 |
| **138** | 712 | -1 | 1 | -1 | -1 | 0 |
| **139** | 699 | 717 | 0.6174 | 717 | 699 | 0.3826 |
| **140** | 721 | -1 | 0.96 | -1 | -1 | 0.0396 |
| **141** | 712 | -1 | 1 | -1 | -1 | 0 |
| **142** | 833 | -1 | 0.83 | 871 | -1 | 0.1638 |
| **143** | 720 | -1 | 0.9996 | 720 | 718 | 0.0004 |
| **144** | 712 | -1 | 1 | -1 | -1 | 0 |
| **145** | 712 | -1 | 1 | -1 | -1 | 0 |
| **146** | 712 | -1 | 0.9992 | -1 | -1 | 0.0008 |
| **147** | 726 | -1 | 0.7892 | -1 | -1 | 0.2108 |
| **148** | 712 | -1 | 0.9968 | 702 | -1 | 0.0032 |
| **149** | 714 | -1 | 0.9914 | 871 | -1 | 0.0072 |
| **150** | 833 | -1 | 0.9998 | -1 | -1 | 0.0002 |
| **151** | 720 | -1 | 0.824 | -1 | -1 | 0.1754 |
| **152** | 834 | 869 | 0.6758 | 869 | 834 | 0.285 |
| **153** | 720 | -1 | 0.9646 | -1 | -1 | 0.0348 |
| **154** | -1 | -1 | 0.9798 | 856 | -1 | 0.0092 |
| **155** | 834 | -1 | 0.575 | -1 | -1 | 0.3232 |
| **156** | 712 | -1 | 0.7902 | -1 | -1 | 0.1964 |
| **157** | 834 | -1 | 0.9692 | 711 | -1 | 0.0178 |
| **158** | 871 | -1 | 0.8268 | -1 | -1 | 0.1652 |
| **159** | 727 | -1 | 0.9458 | -1 | -1 | 0.0542 |
| **160** | 728 | -1 | 1 | -1 | -1 | 0 |
| **161** | 727 | -1 | 0.9968 | -1 | -1 | 0.0032 |
| **162** | 728 | -1 | 0.9994 | 728 | 835 | 0.0004 |
| **163** | 728 | -1 | 0.9998 | -1 | -1 | 0.0002 |
| **164** | 835 | 728 | 0.3612 | 728 | 835 | 0.3232 |
| **165** | 728 | -1 | 1 | -1 | -1 | 0 |
| **166** | 729 | 727 | 0.6894 | 727 | 729 | 0.1656 |
| **167** | 729 | 728 | 0.843 | 728 | 729 | 0.157 |
| **168** | 729 | 724 | 0.9934 | 724 | 729 | 0.0056 |
| **169** | 729 | -1 | 0.9998 | -1 | -1 | 0.0002 |
| **170** | 729 | -1 | 1 | -1 | -1 | 0 |
| **171** | -1 | -1 | 0.6636 | 730 | -1 | 0.2934 |
| **172** | 731 | -1 | 0.9482 | -1 | -1 | 0.0502 |
| **173** | 729 | -1 | 0.8706 | 729 | 707 | 0.1264 |
| **174** | 703 | -1 | 0.7724 | -1 | -1 | 0.1764 |
| **175** | 716 | -1 | 0.9966 | -1 | -1 | 0.0034 |
| **176** | -1 | -1 | 0.9178 | 857 | -1 | 0.0822 |
| **177** | 838 | -1 | 1 | -1 | -1 | 0 |
| **178** | 735 | -1 | 0.9984 | 841 | -1 | 0.0016 |
| **179** | -1 | -1 | 0.9984 | 702 | -1 | 0.0012 |
| **180** | 740 | -1 | 1 | -1 | -1 | 0 |
| **181** | 735 | -1 | 0.9916 | -1 | -1 | 0.0084 |
| **182** | 740 | -1 | 1 | -1 | -1 | 0 |
| **183** | -1 | -1 | 0.9998 | 769 | -1 | 0.0002 |
| **184** | 733 | -1 | 1 | -1 | -1 | 0 |
| **185** | 740 | -1 | 0.8474 | 738 | -1 | 0.129 |
| **186** | 738 | -1 | 0.9966 | -1 | -1 | 0.0034 |
| **187** | 728 | -1 | 0.5682 | 728 | 716 | 0.281 |
| **188** | 733 | -1 | 0.9998 | 733 | 811 | 0.0002 |
| **189** | 738 | -1 | 1 | -1 | -1 | 0 |
| **190** | 764 | -1 | 0.8488 | 738 | -1 | 0.132 |
| **191** | 681 | -1 | 1 | -1 | -1 | 0 |
| **192** | 743 | 682 | 0.8526 | 682 | 743 | 0.1462 |
| **193** | 767 | -1 | 0.5186 | 743 | -1 | 0.4806 |
| **194** | -1 | -1 | 1 | -1 | -1 | 0 |
| **195** | -1 | -1 | 0.9984 | 694 | -1 | 0.0008 |
| **196** | 741 | 743 | 0.3534 | 743 | -1 | 0.3332 |
| **197** | 740 | -1 | 0.9998 | -1 | -1 | 0.0002 |
| **198** | 740 | -1 | 1 | -1 | -1 | 0 |
| **199** | 684 | -1 | 0.8828 | -1 | -1 | 0.1172 |
| **200** | 743 | -1 | 0.9392 | 743 | 826 | 0.0602 |
| **201** | 740 | -1 | 0.9998 | -1 | -1 | 0.0002 |
| **202** | 743 | -1 | 0.9992 | -1 | -1 | 0.0008 |
| **203** | 740 | -1 | 0.5054 | 681 | -1 | 0.4876 |
| **204** | 743 | -1 | 0.9958 | -1 | -1 | 0.0042 |
| **205** | 742 | -1 | 0.9986 | -1 | -1 | 0.0012 |
| **206** | 740 | -1 | 1 | -1 | -1 | 0 |
| **207** | 741 | -1 | 0.9986 | -1 | -1 | 0.0014 |
| **208** | 743 | -1 | 0.9998 | -1 | -1 | 0.0002 |
| **209** | 743 | -1 | 0.9998 | -1 | -1 | 0.0002 |
| **210** | -1 | -1 | 1 | -1 | -1 | 0 |
| **211** | 743 | -1 | 0.814 | 743 | 736 | 0.1808 |
| **212** | 743 | -1 | 1 | -1 | -1 | 0 |
| **213** | 743 | 688 | 0.8664 | 688 | 743 | 0.1336 |
| **214** | 743 | 742 | 0.6676 | 743 | -1 | 0.3054 |
| **215** | 686 | -1 | 0.9506 | 851 | -1 | 0.0486 |
| **216** | 684 | -1 | 0.9922 | -1 | -1 | 0.0078 |
| **217** | 684 | -1 | 0.9072 | -1 | -1 | 0.0928 |
| **218** | 759 | -1 | 0.7914 | 684 | 759 | 0.2036 |
| **219** | 747 | -1 | 0.6646 | -1 | -1 | 0.2874 |
| **220** | -1 | -1 | 0.9792 | 827 | -1 | 0.0104 |
| **221** | -1 | -1 | 0.5296 | 751 | -1 | 0.4704 |
| **222** | 692 | -1 | 0.9992 | -1 | -1 | 0.0006 |
| **223** | 687 | -1 | 0.8698 | 791 | -1 | 0.067 |
| **224** | 680 | 701 | 0.7234 | 701 | 680 | 0.2764 |
| **225** | 766 | -1 | 0.782 | 765 | -1 | 0.1438 |
| **226** | -1 | -1 | 0.981 | 684 | -1 | 0.019 |
| **227** | -1 | -1 | 1 | -1 | -1 | 0 |
| **228** | 687 | -1 | 0.9504 | -1 | -1 | 0.0494 |
| **229** | 749 | 759 | 0.8172 | 759 | 748 | 0.0774 |
| **230** | 743 | -1 | 0.657 | -1 | -1 | 0.343 |
| **231** | -1 | -1 | 0.998 | 684 | -1 | 0.002 |
| **232** | 686 | -1 | 0.999 | 687 | -1 | 0.0006 |
| **233** | 749 | -1 | 0.9618 | 748 | -1 | 0.0346 |
| **234** | 749 | -1 | 0.9678 | 748 | -1 | 0.0322 |
| **235** | 747 | -1 | 0.9844 | -1 | -1 | 0.0156 |
| **236** | 755 | -1 | 0.9966 | -1 | -1 | 0.0034 |
| **237** | 684 | 744 | 0.7424 | 744 | 684 | 0.2574 |
| **238** | 766 | -1 | 0.879 | 765 | -1 | 0.1206 |
| **239** | 749 | -1 | 0.979 | 748 | -1 | 0.021 |
| **240** | 749 | -1 | 0.972 | 748 | -1 | 0.028 |
| **241** | 749 | -1 | 0.9674 | 748 | -1 | 0.0326 |
| **242** | 749 | -1 | 0.9684 | 748 | -1 | 0.0316 |
| **243** | 759 | 810 | 0.9418 | 810 | 759 | 0.056 |
| **244** | 851 | -1 | 0.9854 | -1 | -1 | 0.0118 |
| **245** | 749 | 876 | 0.6126 | 749 | -1 | 0.362 |
| **246** | 763 | 759 | 0.8372 | 759 | 763 | 0.1626 |
| **247** | 785 | 778 | 0.663 | 778 | 785 | 0.3346 |
| **248** | -1 | -1 | 0.8162 | 766 | -1 | 0.165 |
| **249** | 763 | 771 | 0.5198 | 771 | 763 | 0.4776 |
| **250** | 773 | -1 | 0.8698 | -1 | -1 | 0.1294 |
| **251** | 777 | -1 | 0.9884 | -1 | -1 | 0.0116 |
| **252** | 688 | -1 | 0.9734 | -1 | -1 | 0.025 |
| **253** | 684 | -1 | 0.9806 | -1 | -1 | 0.0194 |
| **254** | -1 | -1 | 0.9996 | 871 | -1 | 0.0004 |
| **255** | -1 | -1 | 0.4376 | 764 | -1 | 0.3324 |
| **256** | 766 | -1 | 0.8798 | 765 | -1 | 0.116 |
| **257** | 688 | -1 | 0.9996 | -1 | -1 | 0.0004 |
| **258** | 687 | -1 | 0.9994 | -1 | -1 | 0.0006 |
| **259** | 692 | -1 | 0.9324 | -1 | -1 | 0.0674 |
| **260** | 766 | -1 | 0.8828 | 765 | -1 | 0.1076 |
| **261** | 789 | 786 | 0.8172 | 786 | 789 | 0.1818 |
| **262** | 692 | 694 | 0.8738 | 694 | 692 | 0.1262 |
| **263** | 695 | -1 | 0.9912 | -1 | -1 | 0.0088 |
| **264** | 692 | -1 | 0.973 | -1 | -1 | 0.027 |
| **265** | 687 | -1 | 0.9824 | -1 | -1 | 0.0172 |
| **266** | 689 | -1 | 0.9924 | -1 | -1 | 0.0062 |
| **267** | 766 | -1 | 0.8588 | 765 | -1 | 0.1104 |
| **268** | -1 | -1 | 0.8592 | 703 | -1 | 0.0982 |
| **269** | 749 | -1 | 0.9066 | 748 | -1 | 0.0542 |
| **270** | -1 | -1 | 0.9988 | 744 | -1 | 0.0006 |
| **271** | 688 | -1 | 1 | -1 | -1 | 0 |
| **272** | -1 | -1 | 1 | -1 | -1 | 0 |
| **273** | -1 | -1 | 0.9994 | 703 | -1 | 0.0006 |
| **274** | 812 | -1 | 1 | -1 | -1 | 0 |
| **275** | -1 | -1 | 0.7584 | 809 | -1 | 0.2354 |
| **276** | -1 | -1 | 0.7678 | 801 | -1 | 0.232 |
| **277** | -1 | -1 | 0.9614 | 717 | -1 | 0.0384 |
| **278** | 812 | -1 | 0.981 | 813 | -1 | 0.019 |
| **279** | 812 | -1 | 0.9952 | 766 | -1 | 0.004 |
| **280** | 812 | -1 | 0.682 | 812 | 801 | 0.2918 |
| **281** | 812 | -1 | 1 | -1 | -1 | 0 |
| **282** | 767 | 813 | 0.5612 | 813 | 767 | 0.4364 |
| **283** | 799 | -1 | 0.9974 | -1 | -1 | 0.0026 |
| **284** | 692 | -1 | 0.9874 | -1 | -1 | 0.0122 |
| **285** | -1 | -1 | 0.9988 | 745 | -1 | 0.001 |
| **286** | 812 | -1 | 1 | -1 | -1 | 0 |
| **287** | 767 | -1 | 1 | -1 | -1 | 0 |
| **288** | 812 | 767 | 0.7262 | 767 | 812 | 0.2302 |
| **289** | -1 | -1 | 0.9966 | 824 | -1 | 0.002 |
| **290** | 772 | -1 | 0.9828 | -1 | -1 | 0.0172 |
| **291** | 771 | -1 | 0.989 | -1 | -1 | 0.011 |
| **292** | 771 | -1 | 0.9708 | -1 | -1 | 0.0286 |
| **293** | -1 | -1 | 0.9726 | 772 | -1 | 0.0264 |
| **294** | 787 | -1 | 0.9816 | -1 | -1 | 0.0122 |
| **295** | 789 | -1 | 1 | -1 | -1 | 0 |
| **296** | 724 | -1 | 0.9998 | -1 | -1 | 0.0002 |
| **297** | 692 | -1 | 1 | -1 | -1 | 0 |
| **298** | 763 | -1 | 0.881 | 763 | 762 | 0.1078 |
| **299** | 777 | -1 | 0.9948 | -1 | -1 | 0.0052 |
| **300** | 771 | 772 | 0.5148 | 772 | 771 | 0.4852 |
| **301** | 694 | -1 | 1 | -1 | -1 | 0 |
| **302** | 798 | 767 | 0.5304 | 767 | 798 | 0.4694 |
| **303** | 767 | -1 | 1 | -1 | -1 | 0 |
| **304** | 759 | -1 | 0.994 | 776 | -1 | 0.006 |
| **305** | 789 | -1 | 0.5628 | -1 | -1 | 0.437 |
| **306** | 774 | -1 | 0.9942 | 790 | -1 | 0.0058 |
| **307** | 786 | 771 | 0.635 | 771 | 786 | 0.3648 |
| **308** | 789 | 786 | 0.7196 | 786 | 789 | 0.2096 |
| **309** | 808 | -1 | 0.9994 | -1 | -1 | 0.0006 |
| **310** | 817 | -1 | 0.9896 | -1 | -1 | 0.0104 |
| **311** | 814 | -1 | 0.9232 | -1 | -1 | 0.0766 |
| **312** | 812 | -1 | 0.8444 | -1 | -1 | 0.155 |
| **313** | 812 | -1 | 0.9998 | -1 | -1 | 0.0002 |
| **314** | -1 | -1 | 1 | -1 | -1 | 0 |
| **315** | 819 | -1 | 0.9852 | 820 | -1 | 0.0144 |
| **316** | 812 | -1 | 1 | -1 | -1 | 0 |
| **317** | 810 | -1 | 0.9976 | -1 | -1 | 0.0024 |
| **318** | 814 | -1 | 1 | -1 | -1 | 0 |
| **319** | 815 | -1 | 0.7932 | -1 | -1 | 0.2068 |
| **320** | 814 | -1 | 0.954 | -1 | -1 | 0.0436 |
| **321** | 815 | -1 | 1 | -1 | -1 | 0 |
| **322** | 814 | -1 | 1 | -1 | -1 | 0 |
| **323** | 819 | -1 | 0.7548 | 696 | -1 | 0.1092 |
| **324** | 694 | 818 | 0.4974 | 818 | 694 | 0.4112 |
| **325** | 817 | -1 | 0.9752 | -1 | -1 | 0.0248 |
| **326** | 819 | 697 | 0.6582 | 697 | 819 | 0.3382 |
| **327** | 712 | -1 | 1 | -1 | -1 | 0 |
| **328** | -1 | -1 | 0.9984 | 687 | -1 | 0.0006 |
| **329** | 721 | -1 | 0.99 | 829 | -1 | 0.007 |
| **330** | -1 | -1 | 0.7468 | 873 | -1 | 0.2384 |
| **331** | 832 | -1 | 0.9952 | 871 | -1 | 0.0048 |
| **332** | 871 | -1 | 0.9952 | -1 | -1 | 0.0048 |
| **333** | 833 | -1 | 0.968 | -1 | -1 | 0.0296 |
| **334** | 830 | -1 | 0.999 | 871 | -1 | 0.001 |
| **335** | 699 | 717 | 0.6166 | 717 | 699 | 0.3742 |
| **336** | 833 | -1 | 0.998 | 872 | -1 | 0.0012 |
| **337** | 712 | -1 | 1 | -1 | -1 | 0 |
| **338** | 835 | -1 | 0.9892 | 835 | 878 | 0.0042 |
| **339** | -1 | -1 | 0.9944 | 743 | -1 | 0.0048 |
| **340** | -1 | -1 | 0.6548 | 863 | -1 | 0.265 |
| **341** | 835 | -1 | 0.9998 | -1 | -1 | 0.0002 |
| **342** | -1 | -1 | 0.5066 | 834 | -1 | 0.4904 |
| **343** | 835 | -1 | 0.872 | 832 | -1 | 0.0598 |
| **344** | 871 | -1 | 0.9874 | -1 | -1 | 0.0126 |
| **345** | 718 | 725 | 0.4434 | 725 | -1 | 0.2878 |
| **346** | 873 | -1 | 0.9862 | -1 | -1 | 0.0136 |
| **347** | 727 | -1 | 0.9976 | -1 | -1 | 0.0024 |
| **348** | 835 | -1 | 1 | -1 | -1 | 0 |
| **349** | 834 | -1 | 0.985 | -1 | -1 | 0.008 |
| **350** | 835 | 833 | 0.553 | 833 | 835 | 0.409 |
| **351** | 871 | -1 | 0.5002 | -1 | -1 | 0.4224 |
| **352** | 838 | -1 | 0.9844 | -1 | -1 | 0.0152 |
| **353** | 727 | -1 | 0.6196 | -1 | -1 | 0.3646 |
| **354** | 835 | 838 | 0.9144 | 838 | 835 | 0.0776 |
| **355** | -1 | -1 | 0.9974 | 841 | -1 | 0.0026 |
| **356** | 731 | -1 | 1 | -1 | -1 | 0 |
| **357** | 862 | -1 | 0.9978 | 862 | 718 | 0.0022 |
| **358** | 731 | 838 | 0.7458 | 838 | 731 | 0.2542 |
| **359** | 731 | -1 | 1 | -1 | -1 | 0 |
| **360** | 838 | -1 | 1 | -1 | -1 | 0 |
| **361** | 731 | -1 | 0.9992 | -1 | -1 | 0.0008 |
| **362** | 728 | -1 | 1 | -1 | -1 | 0 |
| **363** | -1 | -1 | 0.9658 | 745 | -1 | 0.0156 |
| **364** | 862 | -1 | 0.991 | 838 | -1 | 0.0086 |
| **365** | 725 | 871 | 0.5218 | 871 | 725 | 0.4482 |
| **366** | -1 | -1 | 0.996 | 878 | -1 | 0.0038 |
| **367** | -1 | -1 | 0.9998 | 703 | -1 | 0.0002 |
| **368** | 857 | -1 | 0.9998 | -1 | -1 | 0.0002 |
| **369** | 835 | -1 | 0.96 | -1 | -1 | 0.04 |
| **370** | 835 | -1 | 0.9874 | -1 | -1 | 0.0126 |
| **371** | -1 | -1 | 0.9506 | 727 | -1 | 0.036 |
| **372** | 835 | -1 | 0.9008 | -1 | -1 | 0.0992 |

| **Prog** | **Mo1** | **Fa1** | **Pr1** | **Mo2** | **Fa2** | **Pr2** |
| --- | --- | --- | --- | --- | --- | --- |
| **1** | 680 | 701 | 0,8564 | 701 | 680 | 0,1436 |
| **2** | 701 | -1 | 0,9864 | -1 | -1 | 0,0136 |
| **3** | -1 | -1 | 0,9998 | 690 | -1 | 0,0002 |
| **4** | 702 | -1 | 0,9046 | -1 | -1 | 0,0954 |
| **5** | 702 | -1 | 0,9648 | -1 | -1 | 0,0348 |
| **6** | 701 | -1 | 0,9984 | -1 | -1 | 0,0016 |
| **7** | 681 | -1 | 0,994 | 705 | -1 | 0,006 |
| **8** | 703 | -1 | 0,9524 | -1 | -1 | 0,044 |
| **9** | 681 | 704 | 0,9714 | 681 | -1 | 0,019 |
| **10** | -1 | -1 | 0,9558 | 705 | -1 | 0,044 |
| **11** | 680 | 825 | 0,9104 | 825 | 680 | 0,081 |
| **12** | 680 | -1 | 0,9996 | 766 | -1 | 0,0002 |
| **13** | 680 | -1 | 1 | -1 | -1 | 0 |
| **14** | 683 | -1 | 1 | -1 | -1 | 0 |
| **15** | 701 | -1 | 0,942 | 683 | -1 | 0,058 |
| **16** | 683 | 684 | 0,679 | 684 | 683 | 0,3208 |
| **17** | 683 | -1 | 0,9986 | -1 | -1 | 0,0014 |
| **18** | 682 | -1 | 1 | -1 | -1 | 0 |
| **19** | 686 | -1 | 0,9994 | -1 | -1 | 0,0006 |
| **20** | 683 | -1 | 1 | -1 | -1 | 0 |
| **21** | 743 | 750 | 0,531 | 743 | -1 | 0,4682 |
| **22** | 743 | -1 | 1 | -1 | -1 | 0 |
| **23** | 684 | 851 | 0,8318 | 851 | 684 | 0,0898 |
| **24** | -1 | -1 | 0,906 | 701 | -1 | 0,094 |
| **25** | 682 | -1 | 0,9998 | -1 | -1 | 0,0002 |
| **26** | -1 | -1 | 0,93 | 800 | -1 | 0,0618 |
| **27** | 687 | -1 | 0,962 | 744 | -1 | 0,0334 |
| **28** | 686 | -1 | 0,6942 | 687 | -1 | 0,2992 |
| **29** | 684 | 740 | 0,8288 | 740 | 684 | 0,1648 |
| **30** | 701 | 691 | 0,8992 | 691 | 701 | 0,1008 |
| **31** | 690 | -1 | 1 | -1 | -1 | 0 |
| **32** | 686 | 684 | 0,6298 | 684 | 686 | 0,2116 |
| **33** | 684 | -1 | 0,6 | -1 | -1 | 0,3998 |
| **34** | 684 | 701 | 0,8322 | 701 | 684 | 0,1676 |
| **35** | 687 | 686 | 0,6354 | 686 | 687 | 0,3624 |
| **36** | 688 | -1 | 0,5344 | 687 | -1 | 0,4376 |
| **37** | 690 | -1 | 0,9356 | 687 | -1 | 0,0644 |
| **38** | 687 | -1 | 0,999 | 791 | -1 | 0,0006 |
| **39** | 687 | -1 | 0,9994 | -1 | -1 | 0,0006 |
| **40** | 690 | -1 | 0,9998 | 687 | -1 | 0,0002 |
| **41** | 687 | -1 | 1 | -1 | -1 | 0 |
| **42** | 688 | 680 | 0,5338 | 680 | 688 | 0,448 |
| **43** | 690 | -1 | 0,999 | -1 | -1 | 0,001 |
| **44** | 688 | -1 | 0,9786 | -1 | -1 | 0,0196 |
| **45** | -1 | -1 | 0,9676 | 835 | -1 | 0,0304 |
| **46** | 688 | -1 | 1 | -1 | -1 | 0 |
| **47** | 812 | -1 | 0,9978 | -1 | -1 | 0,0014 |
| **48** | 686 | -1 | 0,9134 | 701 | -1 | 0,0866 |
| **49** | 819 | -1 | 1 | -1 | -1 | 0 |
| **50** | 812 | -1 | 0,9936 | 688 | -1 | 0,0064 |
| **51** | 724 | -1 | 1 | -1 | -1 | 0 |
| **52** | 694 | -1 | 0,6534 | 692 | -1 | 0,3462 |
| **53** | 812 | -1 | 0,9992 | -1 | -1 | 0,0008 |
| **54** | 743 | -1 | 0,715 | -1 | -1 | 0,2764 |
| **55** | 701 | -1 | 0,9994 | -1 | -1 | 0,0006 |
| **56** | 692 | -1 | 0,9874 | -1 | -1 | 0,01 |
| **57** | 686 | 766 | 0,4164 | 766 | 686 | 0,2604 |
| **58** | 812 | -1 | 0,9994 | -1 | -1 | 0,0004 |
| **59** | 692 | -1 | 0,9992 | -1 | -1 | 0,0006 |
| **60** | 690 | 798 | 0,8244 | 798 | 690 | 0,1744 |
| **61** | 812 | -1 | 1 | -1 | -1 | 0 |
| **62** | 812 | 810 | 0,8994 | 810 | 812 | 0,1006 |
| **63** | -1 | -1 | 0,5016 | 695 | -1 | 0,4982 |
| **64** | 812 | 695 | 0,6896 | 695 | 812 | 0,308 |
| **65** | 812 | -1 | 1 | -1 | -1 | 0 |
| **66** | 694 | 695 | 0,5218 | 695 | 694 | 0,2998 |
| **67** | 812 | -1 | 0,9998 | -1 | -1 | 0,0002 |
| **68** | 812 | -1 | 1 | -1 | -1 | 0 |
| **69** | 694 | -1 | 1 | -1 | -1 | 0 |
| **70** | 812 | -1 | 0,9998 | 817 | -1 | 0,0002 |
| **71** | -1 | -1 | 0,9978 | 801 | -1 | 0,002 |
| **72** | -1 | -1 | 1 | -1 | -1 | 0 |
| **73** | 695 | -1 | 0,653 | -1 | -1 | 0,3412 |
| **74** | 695 | -1 | 0,899 | -1 | -1 | 0,1002 |
| **75** | 819 | 820 | 0,8518 | 820 | 819 | 0,1478 |
| **76** | 717 | -1 | 1 | -1 | -1 | 0 |
| **77** | 819 | 812 | 0,6472 | 812 | 819 | 0,3528 |
| **78** | -1 | -1 | 0,751 | 746 | -1 | 0,2476 |
| **79** | 819 | -1 | 1 | -1 | -1 | 0 |
| **80** | -1 | -1 | 1 | -1 | -1 | 0 |
| **81** | 812 | -1 | 0,9992 | -1 | -1 | 0,0008 |
| **82** | -1 | -1 | 1 | -1 | -1 | 0 |
| **83** | 819 | 695 | 0,694 | 695 | 819 | 0,1426 |
| **84** | 819 | -1 | 0,9998 | -1 | -1 | 0,0002 |
| **85** | -1 | -1 | 0,9992 | 790 | -1 | 0,0004 |
| **86** | 823 | -1 | 1 | -1 | -1 | 0 |
| **87** | 819 | -1 | 0,953 | -1 | -1 | 0,0456 |
| **88** | 697 | -1 | 0,9494 | 724 | -1 | 0,049 |
| **89** | 714 | 718 | 0,5068 | 718 | 714 | 0,4928 |
| **90** | 699 | -1 | 0,8464 | 699 | 730 | 0,1464 |
| **91** | -1 | -1 | 0,97 | 699 | -1 | 0,03 |
| **92** | 819 | 699 | 0,5778 | 699 | 819 | 0,418 |
| **93** | 699 | -1 | 0,9952 | 705 | 699 | 0,0034 |
| **94** | 680 | -1 | 1 | -1 | -1 | 0 |
| **95** | 702 | 701 | 0,71 | 701 | 702 | 0,2892 |
| **96** | -1 | -1 | 0,7078 | 699 | -1 | 0,2922 |
| **97** | 699 | -1 | 1 | -1 | -1 | 0 |
| **98** | -1 | -1 | 1 | -1 | -1 | 0 |
| **99** | 681 | -1 | 0,9998 | -1 | -1 | 0,0002 |
| **100** | -1 | -1 | 0,9996 | 703 | -1 | 0,0004 |
| **101** | 716 | 714 | 0,542 | 714 | 716 | 0,328 |
| **102** | 720 | -1 | 0,9998 | -1 | -1 | 0,0002 |
| **103** | 705 | 706 | 0,6722 | 706 | 705 | 0,3276 |
| **104** | 729 | -1 | 1 | -1 | -1 | 0 |
| **105** | 681 | -1 | 1 | -1 | -1 | 0 |
| **106** | 703 | -1 | 0,8464 | -1 | -1 | 0,0862 |
| **107** | 708 | -1 | 1 | -1 | -1 | 0 |
| **108** | -1 | -1 | 0,9998 | 696 | -1 | 0,0002 |
| **109** | 708 | -1 | 0,9998 | -1 | -1 | 0,0002 |
| **110** | 705 | -1 | 0,9996 | -1 | -1 | 0,0004 |
| **111** | 706 | 727 | 0,7632 | 727 | 706 | 0,2352 |
| **112** | 717 | -1 | 0,9902 | -1 | -1 | 0,0098 |
| **113** | 708 | -1 | 1 | -1 | -1 | 0 |
| **114** | 705 | -1 | 0,992 | 787 | -1 | 0,0054 |
| **115** | 706 | -1 | 0,9996 | -1 | -1 | 0,0004 |
| **116** | 727 | -1 | 1 | -1 | -1 | 0 |
| **117** | -1 | -1 | 0,7282 | 706 | -1 | 0,241 |
| **118** | 817 | -1 | 0,7406 | 700 | -1 | 0,1796 |
| **119** | 729 | -1 | 0,9992 | -1 | -1 | 0,0008 |
| **120** | 836 | 728 | 0,8342 | 728 | 836 | 0,1654 |
| **121** | 835 | -1 | 0,8956 | -1 | -1 | 0,0898 |
| **122** | 727 | -1 | 0,9586 | 728 | -1 | 0,0294 |
| **123** | -1 | -1 | 1 | -1 | -1 | 0 |
| **124** | 712 | -1 | 0,843 | 712 | 835 | 0,1368 |
| **125** | 724 | -1 | 0,9308 | -1 | -1 | 0,069 |
| **126** | 725 | -1 | 0,9374 | 869 | -1 | 0,0614 |
| **127** | 725 | -1 | 1 | -1 | -1 | 0 |
| **128** | 705 | -1 | 0,9854 | -1 | -1 | 0,0146 |
| **129** | 713 | -1 | 0,9998 | -1 | -1 | 0,0002 |
| **130** | -1 | -1 | 0,9996 | 879 | -1 | 0,0004 |
| **131** | -1 | -1 | 1 | -1 | -1 | 0 |
| **132** | 724 | -1 | 1 | -1 | -1 | 0 |
| **133** | 724 | 717 | 0,6288 | 717 | 724 | 0,3702 |
| **134** | 718 | -1 | 0,6694 | -1 | -1 | 0,3284 |
| **135** | 718 | 714 | 0,5068 | 714 | 718 | 0,4932 |
| **136** | 718 | -1 | 0,51 | -1 | -1 | 0,49 |
| **137** | 710 | -1 | 0,7636 | 879 | -1 | 0,1344 |
| **138** | 712 | -1 | 1 | -1 | -1 | 0 |
| **139** | 699 | 717 | 0,6174 | 717 | 699 | 0,3826 |
| **140** | 721 | -1 | 0,96 | -1 | -1 | 0,0396 |
| **141** | 712 | -1 | 1 | -1 | -1 | 0 |
| **142** | 833 | -1 | 0,83 | 871 | -1 | 0,1638 |
| **143** | 720 | -1 | 0,9996 | 720 | 718 | 0,0004 |
| **144** | 712 | -1 | 1 | -1 | -1 | 0 |
| **145** | 712 | -1 | 1 | -1 | -1 | 0 |
| **146** | 712 | -1 | 0,9992 | -1 | -1 | 0,0008 |
| **147** | 726 | -1 | 0,7892 | -1 | -1 | 0,2108 |
| **148** | 712 | -1 | 0,9968 | 702 | -1 | 0,0032 |
| **149** | 714 | -1 | 0,9914 | 871 | -1 | 0,0072 |
| **150** | 833 | -1 | 0,9998 | -1 | -1 | 0,0002 |
| **151** | 720 | -1 | 0,824 | -1 | -1 | 0,1754 |
| **152** | 834 | 869 | 0,6758 | 869 | 834 | 0,285 |
| **153** | 720 | -1 | 0,9646 | -1 | -1 | 0,0348 |
| **154** | -1 | -1 | 0,9798 | 856 | -1 | 0,0092 |
| **155** | 834 | -1 | 0,575 | -1 | -1 | 0,3232 |
| **156** | 712 | -1 | 0,7902 | -1 | -1 | 0,1964 |
| **157** | 834 | -1 | 0,9692 | 711 | -1 | 0,0178 |
| **158** | 871 | -1 | 0,8268 | -1 | -1 | 0,1652 |
| **159** | 727 | -1 | 0,9458 | -1 | -1 | 0,0542 |
| **160** | 728 | -1 | 1 | -1 | -1 | 0 |
| **161** | 727 | -1 | 0,9968 | -1 | -1 | 0,0032 |
| **162** | 728 | -1 | 0,9994 | 728 | 835 | 0,0004 |
| **163** | 728 | -1 | 0,9998 | -1 | -1 | 0,0002 |
| **164** | 835 | 728 | 0,3612 | 728 | 835 | 0,3232 |
| **165** | 728 | -1 | 1 | -1 | -1 | 0 |
| **166** | 729 | 727 | 0,6894 | 727 | 729 | 0,1656 |
| **167** | 729 | 728 | 0,843 | 728 | 729 | 0,157 |
| **168** | 729 | 724 | 0,9934 | 724 | 729 | 0,0056 |
| **169** | 729 | -1 | 0,9998 | -1 | -1 | 0,0002 |
| **170** | 729 | -1 | 1 | -1 | -1 | 0 |
| **171** | -1 | -1 | 0,6636 | 730 | -1 | 0,2934 |
| **172** | 731 | -1 | 0,9482 | -1 | -1 | 0,0502 |
| **173** | 729 | -1 | 0,8706 | 729 | 707 | 0,1264 |
| **174** | 703 | -1 | 0,7724 | -1 | -1 | 0,1764 |
| **175** | 716 | -1 | 0,9966 | -1 | -1 | 0,0034 |
| **176** | -1 | -1 | 0,9178 | 857 | -1 | 0,0822 |
| **177** | 838 | -1 | 1 | -1 | -1 | 0 |
| **178** | 735 | -1 | 0,9984 | 841 | -1 | 0,0016 |
| **179** | -1 | -1 | 0,9984 | 702 | -1 | 0,0012 |
| **180** | 740 | -1 | 1 | -1 | -1 | 0 |
| **181** | 735 | -1 | 0,9916 | -1 | -1 | 0,0084 |
| **182** | 740 | -1 | 1 | -1 | -1 | 0 |
| **183** | -1 | -1 | 0,9998 | 769 | -1 | 0,0002 |
| **184** | 733 | -1 | 1 | -1 | -1 | 0 |
| **185** | 740 | -1 | 0,8474 | 738 | -1 | 0,129 |
| **186** | 738 | -1 | 0,9966 | -1 | -1 | 0,0034 |
| **187** | 728 | -1 | 0,5682 | 728 | 716 | 0,281 |
| **188** | 733 | -1 | 0,9998 | 733 | 811 | 0,0002 |
| **189** | 738 | -1 | 1 | -1 | -1 | 0 |
| **190** | 764 | -1 | 0,8488 | 738 | -1 | 0,132 |
| **191** | 681 | -1 | 1 | -1 | -1 | 0 |
| **192** | 743 | 682 | 0,8526 | 682 | 743 | 0,1462 |
| **193** | 767 | -1 | 0,5186 | 743 | -1 | 0,4806 |
| **194** | -1 | -1 | 1 | -1 | -1 | 0 |
| **195** | -1 | -1 | 0,9984 | 694 | -1 | 0,0008 |
| **196** | 741 | 743 | 0,3534 | 743 | -1 | 0,3332 |
| **197** | 740 | -1 | 0,9998 | -1 | -1 | 0,0002 |
| **198** | 740 | -1 | 1 | -1 | -1 | 0 |
| **199** | 684 | -1 | 0,8828 | -1 | -1 | 0,1172 |
| **200** | 743 | -1 | 0,9392 | 743 | 826 | 0,0602 |
| **201** | 740 | -1 | 0,9998 | -1 | -1 | 0,0002 |
| **202** | 743 | -1 | 0,9992 | -1 | -1 | 0,0008 |
| **203** | 740 | -1 | 0,5054 | 681 | -1 | 0,4876 |
| **204** | 743 | -1 | 0,9958 | -1 | -1 | 0,0042 |
| **205** | 742 | -1 | 0,9986 | -1 | -1 | 0,0012 |
| **206** | 740 | -1 | 1 | -1 | -1 | 0 |
| **207** | 741 | -1 | 0,9986 | -1 | -1 | 0,0014 |
| **208** | 743 | -1 | 0,9998 | -1 | -1 | 0,0002 |
| **209** | 743 | -1 | 0,9998 | -1 | -1 | 0,0002 |
| **210** | -1 | -1 | 1 | -1 | -1 | 0 |
| **211** | 743 | -1 | 0,814 | 743 | 736 | 0,1808 |
| **212** | 743 | -1 | 1 | -1 | -1 | 0 |
| **213** | 743 | 688 | 0,8664 | 688 | 743 | 0,1336 |
| **214** | 743 | 742 | 0,6676 | 743 | -1 | 0,3054 |
| **215** | 686 | -1 | 0,9506 | 851 | -1 | 0,0486 |
| **216** | 684 | -1 | 0,9922 | -1 | -1 | 0,0078 |
| **217** | 684 | -1 | 0,9072 | -1 | -1 | 0,0928 |
| **218** | 759 | -1 | 0,7914 | 684 | 759 | 0,2036 |
| **219** | 747 | -1 | 0,6646 | -1 | -1 | 0,2874 |
| **220** | -1 | -1 | 0,9792 | 827 | -1 | 0,0104 |
| **221** | -1 | -1 | 0,5296 | 751 | -1 | 0,4704 |
| **222** | 692 | -1 | 0,9992 | -1 | -1 | 0,0006 |
| **223** | 687 | -1 | 0,8698 | 791 | -1 | 0,067 |
| **224** | 680 | 701 | 0,7234 | 701 | 680 | 0,2764 |
| **225** | 766 | -1 | 0,782 | 765 | -1 | 0,1438 |
| **226** | -1 | -1 | 0,981 | 684 | -1 | 0,019 |
| **227** | -1 | -1 | 1 | -1 | -1 | 0 |
| **228** | 687 | -1 | 0,9504 | -1 | -1 | 0,0494 |
| **229** | 749 | 759 | 0,8172 | 759 | 748 | 0,0774 |
| **230** | 743 | -1 | 0,657 | -1 | -1 | 0,343 |
| **231** | -1 | -1 | 0,998 | 684 | -1 | 0,002 |
| **232** | 686 | -1 | 0,999 | 687 | -1 | 0,0006 |
| **233** | 749 | -1 | 0,9618 | 748 | -1 | 0,0346 |
| **234** | 749 | -1 | 0,9678 | 748 | -1 | 0,0322 |
| **235** | 747 | -1 | 0,9844 | -1 | -1 | 0,0156 |
| **236** | 755 | -1 | 0,9966 | -1 | -1 | 0,0034 |
| **237** | 684 | 744 | 0,7424 | 744 | 684 | 0,2574 |
| **238** | 766 | -1 | 0,879 | 765 | -1 | 0,1206 |
| **239** | 749 | -1 | 0,979 | 748 | -1 | 0,021 |
| **240** | 749 | -1 | 0,972 | 748 | -1 | 0,028 |
| **241** | 749 | -1 | 0,9674 | 748 | -1 | 0,0326 |
| **242** | 749 | -1 | 0,9684 | 748 | -1 | 0,0316 |
| **243** | 759 | 810 | 0,9418 | 810 | 759 | 0,056 |
| **244** | 851 | -1 | 0,9854 | -1 | -1 | 0,0118 |
| **245** | 749 | 876 | 0,6126 | 749 | -1 | 0,362 |
| **246** | 763 | 759 | 0,8372 | 759 | 763 | 0,1626 |
| **247** | 785 | 778 | 0,663 | 778 | 785 | 0,3346 |
| **248** | -1 | -1 | 0,8162 | 766 | -1 | 0,165 |
| **249** | 763 | 771 | 0,5198 | 771 | 763 | 0,4776 |
| **250** | 773 | -1 | 0,8698 | -1 | -1 | 0,1294 |
| **251** | 777 | -1 | 0,9884 | -1 | -1 | 0,0116 |
| **252** | 688 | -1 | 0,9734 | -1 | -1 | 0,025 |
| **253** | 684 | -1 | 0,9806 | -1 | -1 | 0,0194 |
| **254** | -1 | -1 | 0,9996 | 871 | -1 | 0,0004 |
| **255** | -1 | -1 | 0,4376 | 764 | -1 | 0,3324 |
| **256** | 766 | -1 | 0,8798 | 765 | -1 | 0,116 |
| **257** | 688 | -1 | 0,9996 | -1 | -1 | 0,0004 |
| **258** | 687 | -1 | 0,9994 | -1 | -1 | 0,0006 |
| **259** | 692 | -1 | 0,9324 | -1 | -1 | 0,0674 |
| **260** | 766 | -1 | 0,8828 | 765 | -1 | 0,1076 |
| **261** | 789 | 786 | 0,8172 | 786 | 789 | 0,1818 |
| **262** | 692 | 694 | 0,8738 | 694 | 692 | 0,1262 |
| **263** | 695 | -1 | 0,9912 | -1 | -1 | 0,0088 |
| **264** | 692 | -1 | 0,973 | -1 | -1 | 0,027 |
| **265** | 687 | -1 | 0,9824 | -1 | -1 | 0,0172 |
| **266** | 689 | -1 | 0,9924 | -1 | -1 | 0,0062 |
| **267** | 766 | -1 | 0,8588 | 765 | -1 | 0,1104 |
| **268** | -1 | -1 | 0,8592 | 703 | -1 | 0,0982 |
| **269** | 749 | -1 | 0,9066 | 748 | -1 | 0,0542 |
| **270** | -1 | -1 | 0,9988 | 744 | -1 | 0,0006 |
| **271** | 688 | -1 | 1 | -1 | -1 | 0 |
| **272** | -1 | -1 | 1 | -1 | -1 | 0 |
| **273** | -1 | -1 | 0,9994 | 703 | -1 | 0,0006 |
| **274** | 812 | -1 | 1 | -1 | -1 | 0 |
| **275** | -1 | -1 | 0,7584 | 809 | -1 | 0,2354 |
| **276** | -1 | -1 | 0,7678 | 801 | -1 | 0,232 |
| **277** | -1 | -1 | 0,9614 | 717 | -1 | 0,0384 |
| **278** | 812 | -1 | 0,981 | 813 | -1 | 0,019 |
| **279** | 812 | -1 | 0,9952 | 766 | -1 | 0,004 |
| **280** | 812 | -1 | 0,682 | 812 | 801 | 0,2918 |
| **281** | 812 | -1 | 1 | -1 | -1 | 0 |
| **282** | 767 | 813 | 0,5612 | 813 | 767 | 0,4364 |
| **283** | 799 | -1 | 0,9974 | -1 | -1 | 0,0026 |
| **284** | 692 | -1 | 0,9874 | -1 | -1 | 0,0122 |
| **285** | -1 | -1 | 0,9988 | 745 | -1 | 0,001 |
| **286** | 812 | -1 | 1 | -1 | -1 | 0 |
| **287** | 767 | -1 | 1 | -1 | -1 | 0 |
| **288** | 812 | 767 | 0,7262 | 767 | 812 | 0,2302 |
| **289** | -1 | -1 | 0,9966 | 824 | -1 | 0,002 |
| **290** | 772 | -1 | 0,9828 | -1 | -1 | 0,0172 |
| **291** | 771 | -1 | 0,989 | -1 | -1 | 0,011 |
| **292** | 771 | -1 | 0,9708 | -1 | -1 | 0,0286 |
| **293** | -1 | -1 | 0,9726 | 772 | -1 | 0,0264 |
| **294** | 787 | -1 | 0,9816 | -1 | -1 | 0,0122 |
| **295** | 789 | -1 | 1 | -1 | -1 | 0 |
| **296** | 724 | -1 | 0,9998 | -1 | -1 | 0,0002 |
| **297** | 692 | -1 | 1 | -1 | -1 | 0 |
| **298** | 763 | -1 | 0,881 | 763 | 762 | 0,1078 |
| **299** | 777 | -1 | 0,9948 | -1 | -1 | 0,0052 |
| **300** | 771 | 772 | 0,5148 | 772 | 771 | 0,4852 |
| **301** | 694 | -1 | 1 | -1 | -1 | 0 |
| **302** | 798 | 767 | 0,5304 | 767 | 798 | 0,4694 |
| **303** | 767 | -1 | 1 | -1 | -1 | 0 |
| **304** | 759 | -1 | 0,994 | 776 | -1 | 0,006 |
| **305** | 789 | -1 | 0,5628 | -1 | -1 | 0,437 |
| **306** | 774 | -1 | 0,9942 | 790 | -1 | 0,0058 |
| **307** | 786 | 771 | 0,635 | 771 | 786 | 0,3648 |
| **308** | 789 | 786 | 0,7196 | 786 | 789 | 0,2096 |
| **309** | 808 | -1 | 0,9994 | -1 | -1 | 0,0006 |
| **310** | 817 | -1 | 0,9896 | -1 | -1 | 0,0104 |
| **311** | 814 | -1 | 0,9232 | -1 | -1 | 0,0766 |
| **312** | 812 | -1 | 0,8444 | -1 | -1 | 0,155 |
| **313** | 812 | -1 | 0,9998 | -1 | -1 | 0,0002 |
| **314** | -1 | -1 | 1 | -1 | -1 | 0 |
| **315** | 819 | -1 | 0,9852 | 820 | -1 | 0,0144 |
| **316** | 812 | -1 | 1 | -1 | -1 | 0 |
| **317** | 810 | -1 | 0,9976 | -1 | -1 | 0,0024 |
| **318** | 814 | -1 | 1 | -1 | -1 | 0 |
| **319** | 815 | -1 | 0,7932 | -1 | -1 | 0,2068 |
| **320** | 814 | -1 | 0,954 | -1 | -1 | 0,0436 |
| **321** | 815 | -1 | 1 | -1 | -1 | 0 |
| **322** | 814 | -1 | 1 | -1 | -1 | 0 |
| **323** | 819 | -1 | 0,7548 | 696 | -1 | 0,1092 |
| **324** | 694 | 818 | 0,4974 | 818 | 694 | 0,4112 |
| **325** | 817 | -1 | 0,9752 | -1 | -1 | 0,0248 |
| **326** | 819 | 697 | 0,6582 | 697 | 819 | 0,3382 |
| **327** | 712 | -1 | 1 | -1 | -1 | 0 |
| **328** | -1 | -1 | 0,9984 | 687 | -1 | 0,0006 |
| **329** | 721 | -1 | 0,99 | 829 | -1 | 0,007 |
| **330** | -1 | -1 | 0,7468 | 873 | -1 | 0,2384 |
| **331** | 832 | -1 | 0,9952 | 871 | -1 | 0,0048 |
| **332** | 871 | -1 | 0,9952 | -1 | -1 | 0,0048 |
| **333** | 833 | -1 | 0,968 | -1 | -1 | 0,0296 |
| **334** | 830 | -1 | 0,999 | 871 | -1 | 0,001 |
| **335** | 699 | 717 | 0,6166 | 717 | 699 | 0,3742 |
| **336** | 833 | -1 | 0,998 | 872 | -1 | 0,0012 |
| **337** | 712 | -1 | 1 | -1 | -1 | 0 |
| **338** | 835 | -1 | 0,9892 | 835 | 878 | 0,0042 |
| **339** | -1 | -1 | 0,9944 | 743 | -1 | 0,0048 |
| **340** | -1 | -1 | 0,6548 | 863 | -1 | 0,265 |
| **341** | 835 | -1 | 0,9998 | -1 | -1 | 0,0002 |
| **342** | -1 | -1 | 0,5066 | 834 | -1 | 0,4904 |
| **343** | 835 | -1 | 0,872 | 832 | -1 | 0,0598 |
| **344** | 871 | -1 | 0,9874 | -1 | -1 | 0,0126 |
| **345** | 718 | 725 | 0,4434 | 725 | -1 | 0,2878 |
| **346** | 873 | -1 | 0,9862 | -1 | -1 | 0,0136 |
| **347** | 727 | -1 | 0,9976 | -1 | -1 | 0,0024 |
| **348** | 835 | -1 | 1 | -1 | -1 | 0 |
| **349** | 834 | -1 | 0,985 | -1 | -1 | 0,008 |
| **350** | 835 | 833 | 0,553 | 833 | 835 | 0,409 |
| **351** | 871 | -1 | 0,5002 | -1 | -1 | 0,4224 |
| **352** | 838 | -1 | 0,9844 | -1 | -1 | 0,0152 |
| **353** | 727 | -1 | 0,6196 | -1 | -1 | 0,3646 |
| **354** | 835 | 838 | 0,9144 | 838 | 835 | 0,0776 |
| **355** | -1 | -1 | 0,9974 | 841 | -1 | 0,0026 |
| **356** | 731 | -1 | 1 | -1 | -1 | 0 |
| **357** | 862 | -1 | 0,9978 | 862 | 718 | 0,0022 |
| **358** | 731 | 838 | 0,7458 | 838 | 731 | 0,2542 |
| **359** | 731 | -1 | 1 | -1 | -1 | 0 |
| **360** | 838 | -1 | 1 | -1 | -1 | 0 |
| **361** | 731 | -1 | 0,9992 | -1 | -1 | 0,0008 |
| **362** | 728 | -1 | 1 | -1 | -1 | 0 |
| **363** | -1 | -1 | 0,9658 | 745 | -1 | 0,0156 |
| **364** | 862 | -1 | 0,991 | 838 | -1 | 0,0086 |
| **365** | 725 | 871 | 0,5218 | 871 | 725 | 0,4482 |
| **366** | -1 | -1 | 0,996 | 878 | -1 | 0,0038 |
| **367** | -1 | -1 | 0,9998 | 703 | -1 | 0,0002 |
| **368** | 857 | -1 | 0,9998 | -1 | -1 | 0,0002 |
| **369** | 835 | -1 | 0,96 | -1 | -1 | 0,04 |
| **370** | 835 | -1 | 0,9874 | -1 | -1 | 0,0126 |
| **371** | -1 | -1 | 0,9506 | 727 | -1 | 0,036 |
| **372** | 835 | -1 | 0,9008 | -1 | -1 | 0,0992 |
